# Mechanoadaptive organization of stress fiber subtypes in epithelial cells under cyclic stretches and stretch release

**DOI:** 10.1038/s41598-020-75791-2

**Published:** 2020-10-29

**Authors:** Amir Roshanzadeh, Tham Thi Nguyen, Khoa Dang Nguyen, Dong-Su Kim, Bong-Kee Lee, Dong-Weon Lee, Eung-Sam Kim

**Affiliations:** 1 School of Biological Sciences and Biotechnology, Chonnam National University, Gwangju, Republic of Korea; 2 Department of Mechanical Engineering, Chonnam National University, Gwangju, Republic of Korea; 3 Department of Biological Sciences and Research Center of Ecomimetics, Chonnam National University, Gwangju, 61186 Republic of Korea; 4 Center for Next Generation Sensor Research and Development, Chonnam National University, Gwangju, Republic of Korea

**Keywords:** Cytoskeleton, Stress fibres, Cellular motility

## Abstract

Cyclic stretch applied to cells induces the reorganization of stress fibers. However, the correlation between the reorganization of stress fiber subtypes and strain-dependent responses of the cytoplasm and nucleus has remained unclear. Here, we investigated the dynamic involvement of stress fiber subtypes in the orientation and elongation of cyclically stretched epithelial cells. We applied uniaxial cyclic stretches at 5%, 10%, and 15% strains to cells followed by the release of the mechanical stretch. Dorsal, transverse arcs, and peripheral stress fibers were mainly involved in the cytoplasm responses whereas perinuclear cap fibers were associated with the reorientation and elongation of the nucleus. Dorsal stress fibers and transverse arcs rapidly responded within 15 min regardless of the strain magnitude to facilitate the subsequent changes in the orientation and elongation of the cytoplasm. The cyclic stretches induced the additional formation of perinuclear cap fibers and their increased number was almost maintained with a slight decline after 2-h-long stretch release. The slow formation and high stability of perinuclear cap fibers were linked to the slow reorientation kinetics and partial morphology recovery of nucleus in the presence or absence of cyclic stretches. The reorganization of stress fiber subtypes occurred in accordance with the reversible distribution of myosin II. These findings allowed us to propose a model for stretch-induced responses of the cytoplasm and nucleus in epithelial cells based on different mechanoadaptive properties of stress fiber subtypes.

## Introduction

The organization of stress fibers (SFs) in response to applied strains is important for understanding mechanotransduction and mechanohomeostasis of cells or tissues. The SF organization is a structural adaptation that allows mechanically stretched cells to adjust their shapes and orientations^1–3^. The application of mechanical cues to mammalian cells has revealed rapid changes in SFs compared to other cytoskeletal components such as microtubules and intermediate filaments. It has been known that stretch-induced reorientation and elongation of cells are microtubule-independent while the destabilization of SFs significantly inhibits cellular mechanoadpatations^4–6^.

SFs that are distributed at different cellular locations with different structures can be divided into four subtypes: perinuclear cap fiber, dorsal stress fiber, transverse arc, and ventral stress fiber. Among them, perinuclear cap fibers are connected to the apical surface of the nucleus through LINC (linker of nucleoskeleton and cytoskeleton) complex and to the extracellular matrix (ECM) through their focal adhesion complex (FAC), allowing the nucleus to sense and response to mechanical cues from ECM^7–9^. In contrast to perinuclear cap fibers, the other SF subtypes are not directly connected to the nucleus. However, the interconnection of dorsal stress fibers, ventral stress fibers, and transverse arcs could induce the formation of perinuclear cap fibers and subsequently control the nuclear movement in migrating cells^9,10^. Dorsal stress fibers are devoid of myosin II in the uniform polarity of their structure suggesting the non-contractile properties. They are polymerized at one end that is attached to FAC, whereas the other end is connected to transverse arcs (curved-shape SF subtypes) which exhibit the typical contractile properties of actomyosin bundles^11–14^. Through these mechanical coupling, the contractility of transverse arcs generates the pulling forces to dorsal stress fibers that induce the FAC maturation^14^. In contrast to non-contractile dorsal stress fibers, ventral stress fibers exhibit the opposite polarity architecture with the incorporation of myosin II^12^. A member of ventral stress fibers is peripheral stress fibers which are composed of thick long bundle of stress fibers and located at the periphery of the cell. These fibers generate the protruding and non-protruding regions in migratory cells^15^. Peripheral stress fibers also exert higher contractile forces than do perinuclear cap fibers^16^.

There are various observations showing the strain-dependent characteristics of cell reorientation in the presence of mechanical stretches: the percentage of reoriented cells is proportional to the magnitude of the strain^1–3^; the final orientation of stretched cells on flexible membranes is determined by the combination of tensile and compressive strains^2,17^. These studies have clarified the crucial role of the SF reorganization in maintaining cellular mechanohomeostasis in cell-stretching conditions.

Although the overall reorientation of stretched cells has been reported, the kinetic response of the cytoplasm is not distinguished from that of nucleus yet. Since the nuclear membrane can be linked with perinuclear cap fibers which are produced by other SFs in the cytoplasm in response to mechanical stretches, it can be expected that these two kinetics may be highly interdependent to coordinate the overall cell reorientation. Few studies focused on their recovery kinetics after removal or release of mechanical stretches to cells. Furthermore, how each subtype of SFs is reorganized or remodeled to give morphological changes and reorientation of the cytoplasm and nucleus has been elusive. Given that stress fibers play a crucial role in strain-dependent responses and most cells in the multi-cellular organisms are exposed to dynamic strains rather than static strains, we assume that the SF subtypes may show different mechanosensitive properties to fine-tune strain-dependent kinetics in reorientation and morphological changes of the cytoplasm and nucleus of cyclically stretched or stretch-released cells.

Recently, embryonic stem cells (ESCs) and induced pluripotent stem cells (iPSCs) have been differentiated into human alveolar epithelial type I (ATI) or type II (ATII) cells^18,19^. However, the low yield and in vitro transition into ATI cells of ATII cells led us to choose A549 cell line as our study model although its origin is an ATII adenocarcinoma. In this study, we applied uniaxial cyclic stretches at 5%, 10%, and 15% strains to A549 cells (a cell line derived from human alveolar epithelium) and analyzed the kinetic profiles of CS-induced changes in reorientation and morphology of the cytoplasm and nucleus. A549 cells grown on the flexible and transparent polymer membrane were cyclically stretched every 3 s (0.3 Hz) for 2 h and then released from the dynamic stretch in an on-stage cell incubator to track the cellular responses by time-lapse imaging. Confocal z-stack imaging was employed to obtain the spatio-temporal localization of SF subtypes and focal adhesion complex. The strain-dependent reorganization of the SF subtypes was correlated with the distribution of myosin II. Our findings enabled us to propose a model for the interdependency of SF subtypes for the mechnoadaptive remodeling of the cytoplasm and nucleus of epithelial cells in the presence or release of cyclic stretches.

## Materials and methods

### Materials

All reagent-grade chemicals, including blebbistatin, cucurbitacin E, ethanol, Triton X-100, and formaldehyde were purchased from Sigma-Aldrich (St. Louis, MO, USA) unless otherwise mentioned. RPMI1640 (Cat. No. SH30027.01, Hyclone) supplemented with l-glutamine was obtained from GE Healthcare Life Sciences (Pittsburgh, PA, USA). Hoechst 33342, a nucleic acid stain dye, was obtained from Invitrogen (Waltham, MA, USA). Fibronectin extracellular matrix protein (Cat. No. 356008, Corning) was purchased from Life Sciences (Corning, NY, USA).

### Cell stretching system

CS was applied to the cells according to a previously described method^2,17^. The lab-made tension system consisted of four parts: stage-top incubator [Live Cell Instrument (LCI)], force regulator to control tension forces, CO_2_ and humidity (LCI CU-501), and a computer to control strain profiles applied to polydimethylsiloxane (PDMS) well. Cells were exposed to uniaxial CS at three different magnitudes, 5%, 10%, and 15%, at 0.3 Hz frequency for 15, 30, 60, and 120 min (Supplementary Fig. S1B).

### PDMS well fabrication

Prepolymer solution of PDMS was prepared at a 10:1 (w/w) ratio of base polymer to crosslinker (Sylgard 184, Dow Corning). The PDMS solution was then vacuum-treated in a desiccator for 30 min to remove bubbles. Subsequently, the mixed PDMS solution was poured onto the assembled PDMS cell-culture well that was composed of two parts, including a flat bottom and a rectangular sidewall (Supplementary Fig. S1A). Further, the PDMS was cured at 80 °C for 1 h and 30 min. The assembled flat bottom and sidewall of PDMS membranes were bonded through an oxygen-based atmospheric plasma treatment (CUTE-1MPR, Femto Science Inc.) at 100 W for a fixed period of 60 s. A linear model of the finite element method (FEM) was applied to analyze the mechanical properties of PDMS well-stretched at 5%, 10%, and 15% (Supplementary Fig. S1C). PDMS deformation was considered in the linear region under 15% strain^20^.

### Cell culture

A549 cells (human lung alveolar epithelial cells from Korean Cell Line Bank) were cultured in an incubator at 37 °C and 5% CO_2_ in growth RPMI1640 medium supplemented with 10% fetal bovine serum (FBS), 100 U/mL penicillin, and 100 mg/mL streptomycin. PDMS substrates were sterilized using 70% ethanol and treated with oxygen plasma (100 W in power) for a fixed period of 60 s. The plasma pretreated PDMS membranes were coated with 114 nM fibronectin in Dulbecco’s phosphate-buffered saline for 2 h at 37 °C. Subsequently, 2.5 × 10^5^ cells/mL were cultured in a PDMS well [4 cm (length) × 2.4 cm (width) × 3.5 cm (height)] for 24 h. Cells were subjected to cyclic strains (5%, 10%, and 15% at 0.3 Hz) for 2 h and fixed immediately afterward.

### Orientation angle of cell body nucleus and FAC

The ellipse model was fitted into the cell and nucleus. The major and minor axes of each cell were manually determined using bright field cell images. The orientation angle was measured between the major axes of the cell body, nucleus, and FAC in response to stretch direction. Orientation angels of the cell body and nucleus were in the range of 0° to 90°^17^, whereas the angle of FAC was in the range of 0° to 360°. For large-scale quantification, the orientation angle was measured using ImageJ v.1.52a software (National Institutes of Health, USA). The cell body and nucleus orientation angles were subsequently presented in boxplots with whisker of boxes corresponding to 5 and 95 percentiles, and the middle line presented a median value; the distribution angle of FAC was presented by polar r theta, where theta is the angle and r is the radius.

### Cell body and nuclear morphology

To evaluate the effects of uniaxial CS to the cell body and nucleus morphology, area and elongation parameters were calculated using ImageJ (v.1.52a) software. Phase contrast images were imported to ImageJ software and the brightness/contrast of images were manually adjusted to extract the cellular morphology (Supplementary Fig. S2). The area was computed based upon the ellipse area equation with the major and minor axes. Elongation parameter was determined as the ratio of the difference to the sum of the major and minor axes according to a previous study^6^. When the elongation parameter is equal to 0, the cell shape is considered completely circular. The elongation parameter for cells showing straight morphology is close to 1.

### Extraction of time constants from kinetic profiles

Two exponential models, including an exponential increase between the limits and an exponential decrease between the limits corresponding to the equations: Y = Y_o_ + (plateau-Y_o_) × ($1-{e}^{-\frac{x}{\tau }} )$ and Y = (Y_o_ − plateau) × ${e}^{-\frac{x}{\tau }}$+ plateau, respectively, were applied for the data analysis. Y_o_ is the value when x (time) is zero, the plateau is the Y value at infinite time, and τ is the time constant, expressed in minute. The time constant represents how rapid the process occurred.

### Immunofluorescence staining

The A549 cells cultured in the PDMS well were fixed in 4% formaldehyde solution for 15 min at 25 °C and washed thrice using phosphate-buffered saline (PBS). Thereafter, permeabilization was accomplished using 0.2% Triton X-100 (Cat. No. T8787, Sigma-Aldrich) in PBS for 15 min at 25 °C. The samples were further incubated in blocking solution using 3% bovine serum albumin (BSA) for 1 h at 25 °C. The primary antibodies, vinculin (Cat. No. ab129002, Abcam, 1:250), myosin IIa (Cat. No. 3403, Cell Signaling Technology, 1:50), and F-actin probe conjugated to the rhodamine-phalloidin (Cat. No. R415, Invitrogen) were diluted in 1% BSA for 1.5 h at 25 °C. The secondary antibody, Alexa-Fluor-488 goat-anti rabbit IgG (Cat. No. A11304, Invitrogen, 1:200) was diluted in the same blocking solution and incubated for 2 h at 25 °C. Finally, the PDMS membrane was mounted onto glass slides using ProLong Gold antifade reagent with DAPI, a nucleic acid stain dye (Cat. No. P6931, Invitrogen).

### Fluorescence microscopy

Z-stack images were acquired using a laser confocal scanning microscope (TCS SP5 AOBS/TANDOM, Leica Microsystems, Germany) equipped with an HCX PL APO × 63 oil-immersion objective lens. The subtype stress fibers and conformational changes of myosin II were analyzed using LAS-AF software (Ver. 2.3.5). Nuclear/stress fibers morphometric features, including elongation parameter and area, and reorientation of cells were acquired using a Lionheart LFX microscopy (BioTek).

### Number of stress fiber subtypes

The SF subtypes were distinguished using fluorescently labeled actin SF(rhodamine-phalloidin) and focal adhesion molecules (vinculin)^12^. The number of each subpopulation of stress fibers per cell was determined through the manual counting of dorsal, ventral, and transverse arcs under indicated conditions. The SF subtypes were distinguished based on their location and connection to focal adhesion complex (FAC). Dorsal SFs were connected to FAC and transverse arcs at their proximal and distal ends, respectively, while transverse arcs were not directly attached to FAs and usually formed in parallel bundles. The peripheral SFs are located at the cell periphery and perinuclear cap fibers that are positioned over the nucleus.

### Inhibition of myosin II

Cells were pre-incubated with blebbistatin (50 µM) for 1 h at 37 °C. Further, cells were subjected to 15% CS at 0.3 Hz with/or without the washing of inhibitor at an indicated time point. The effect of blebbistatin and cyclic stretch were further analyzed through immunocytochemistry analysis.

### F-actin stabilizing

To investigate the cucurbitacin E’s (CuE) effects on actin filaments, A549 cells were pre-incubated with CuE at 10 nM for 1 h. Further, cells were subjected to 15% CS at 0.3 Hz in the presence of the inhibitor for indicated time points. The effect of CuE and cyclic stretch on cell reorientation and SF reorganization was further examined through immunocytochemistry analysis.

### Myosin band spacing

The structure of contractile stress fibers was characterized through the analysis of periodic myosin II bands (Supplementary Fig. S3). Confocal microphotographs of fluorescently labeled myosin II were applied to calculate the average distance between neighboring myosin bands using LAS-AF software (ver. 2.3.5)^21^.

### Cell viability

To determine the cell viability, A549 cells were seeded at 2.5 × 10^5^ cells/PDMS well. Next day, cells were subjected to cyclic stretch with/or without blebbistatin at the indicated time points (0, 15, 30, 60, and 120 min). Cell viability was evaluated after 24 h of post-cyclic stretch using a LIVE/DEAD viability/cytotoxicity kit (Cat. No. BDA-1000, Biomax) following the manufacturer’s instructions. The cell viability was measured by incubating cells with 1 mL of working Live/Dead stains at 37 °C and 5% CO_2_ for 30 min. Subsequently, the cells were washed with PBS and images were captured using the LFX microscope (BioTek). Cell viability was quantified by calculating scores for live (green) and dead (red) from three random fields of PDMS membrane and calculated using Gene 5 software (BioTek).

### Western blotting

Equal amounts of 40 μg total protein were separated on 8% sodium dodecyl sulfate–polyacrylamide gel and electrotransferred onto nitrocellulose membranes. The membranes were washed with Tris‐buffered saline (10 mmol L^−1^ Tris HCl, 150 mmol L^−1^ NaCl, pH 7.5) supplemented with 0.05% (v/v) Tween 20 (TBST) followed by blocking using TBST containing 5% (w/v) skimmed milk. The membranes were incubated overnight with specific primary antibodies to FA kinase (FAK) (Cat. No. 13009, Cell Signaling Technology, 1:1000), phospho-FAK (Cat. No. 3283, Cell Signaling Technology, 1:1000), paxillin (Cat. No. D9G12, Cell Signaling Technology, 1:1000), phospho-paxillin (Cat. No. 2541, Cell Signaling Technology, 1:1000), Myosin IIa (Cat. No. 3403S, Cell Signaling Technology, 1:1000), and β-actin (Cat. No. MA5-15739, Invitrogen, dilution factor 1: 10,000) at 4 °C. The membranes were exposed to secondary antibodies conjugated to horseradish peroxidase for 1 h at 25 °C and treated with ECL reagents. Since the target proteins investigated here are well known cytoskeleton-associated proteins, therefore, we cut the nitrocellulose membrane according to their expected band sizes as shown in Supplementary Fig. S8.

### Statistical analysis

Data are presented as the mean ± SD for at least three independent experiments. One-way analysis of variance was employed to analyze the statistical data among different groups. *p* < 0.01 was considered statistically significant.

## Results

### Set-up for cyclically stretching cells and imaging stress fibers

**Figure 1:**
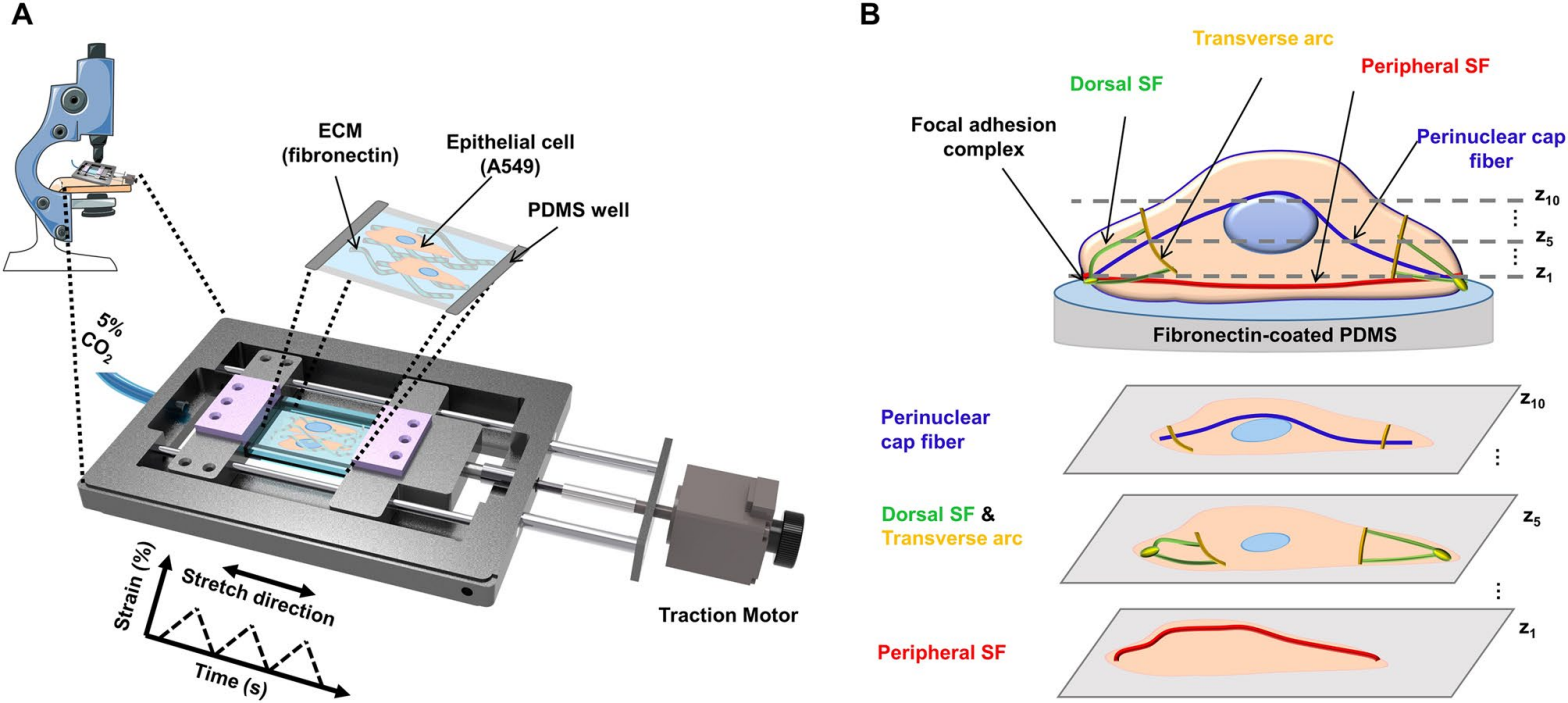
Scheme of a cell-stretching device and stress fiber classification for epithelial cells. (**A**) A549 cells were cultured in a polydimethylsiloxane (PDMS) well and subjected to uniaxial cyclic stretch (CS) in a stage-top cell stretching device. (**B**) Stress fiber (SF) subtypes were classified into four groups: perinuclear cap fibers, transverse arcs, dorsal SFs, and peripheral SFs. The morphological changes and SF subtypes are distinguished using z-stack images of single cells that were obtained through confocal laser scanning microscopy.

To monitor the dynamic responses of epithelial cells to different strains of cyclic stretches, we built a uniaxial tension device in which a PDMS well was cyclically stretched in an on-stage cell incubator of an optical microscope (Fig. 1A). A549 cells, the cell line from the alveolar epithelium of the human lung, were seeded onto a fibronectin-coated PDMS membrane and mechanically stretched for designated time points at 0.3 Hz with peak strains of 5%, 10%, and 15% followed by the release of cyclic stretches. The typical strain found in human alveoli is ranged from 4 to 12% strain in the normal breathing condition^22^. The duration of our cyclic stretches was limited to 2 h since 10% of stretched cells at 15% strain for 2 h were found to be apoptotic after 24 h (Supplementary Fig. S4A,B). We could see no more change in cellular reorientation in response to 10%-strained stretch after 1 h. The triangular strain profile (Supplementary Fig. S1B) was applied to the PDMS well by a computer-controlled traction motor. Cells were cultured at 37 °C and 5% CO_2_ in a humidity-controlled on-stage incubator. The condensation on the lid of the on-stage incubator was negligible through electrically heating the lid, which allowed facile cell monitoring. As illustrated in Fig. 1B, the four SF subtypes were distinguished through the staining of F-actin, vinculin, and myosin II in unstretched or cyclically stretched cells. To visualize the morphological changes and three-dimensional reorganization of SF subtypes in response to cyclic stretches, the z-stack images of single cells were obtained through confocal fluorescence microscopy (Supplementary Fig. S5).

### Strain-dependent reorientation and morphology of the cytoplasm and nucleus of epithelial cells under cyclic stretches followed by stretch relaxation

**Figure 2:**
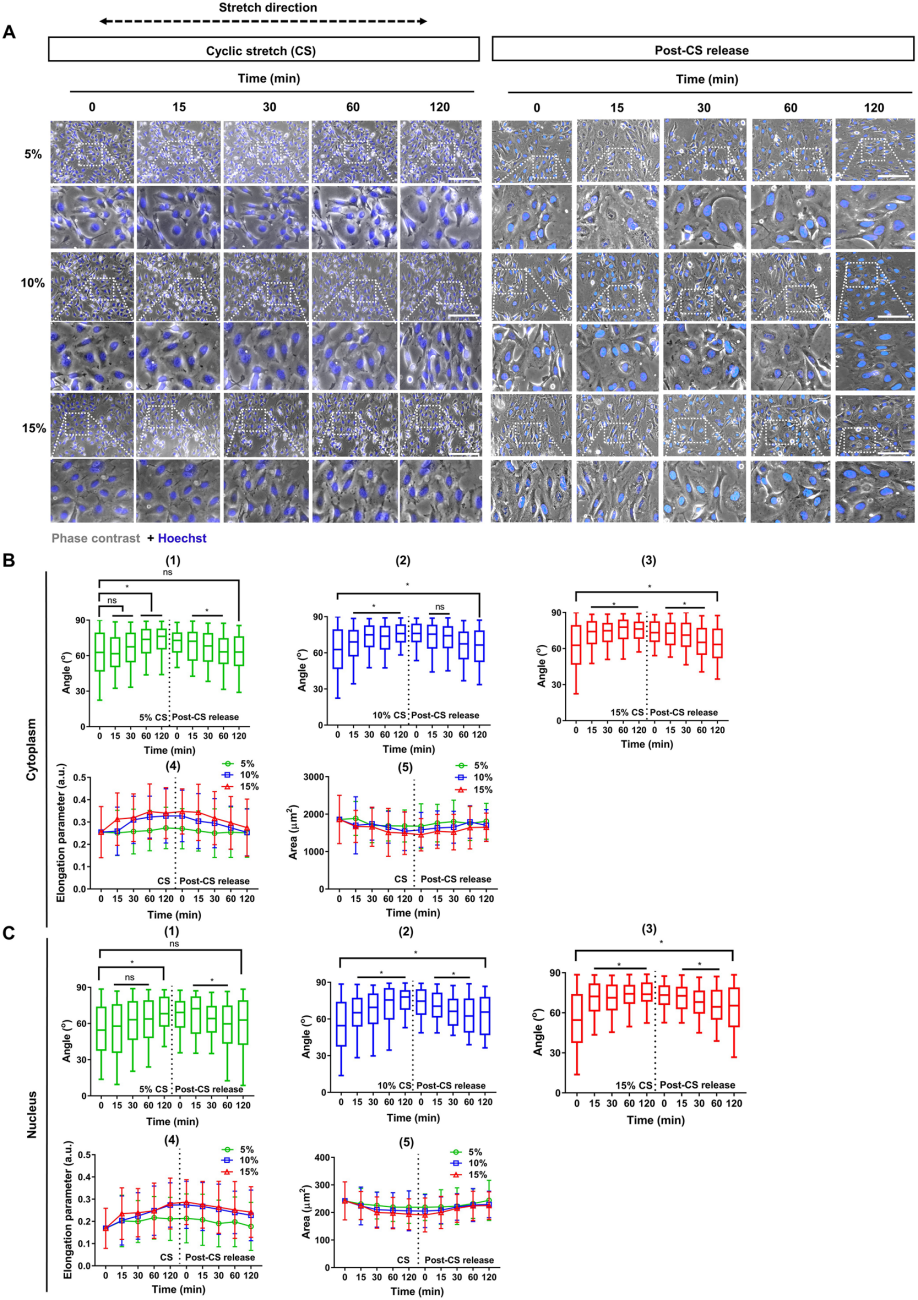
Microscopic images and quantitative analysis of A549 cells in response to cyclic stretches. (**A**) Representative microscopic images of the cell body (phase contrast) and nucleus (Hoechst) after uniaxial cyclic stretch (CS) at 5%, 10%, and 15% strains for the indicated time points. The time kinetic reorientation of cells in the presence of CS and post-CS release is shown in the left and right panels, respectively. Scale bars: 100 µm. (**B**) Kinetic profiles of the reorientation angle, elongation parameter, of the cell bodies in cells subjected to CS and post-CS release (n = 200). (**C**) Kinetic profiles of the reorientation angle, elongation parameter, of the cell nuclei in cells subjected to CS and post-CS release (n = 200). Reorientation data are presented in the box–whisker plot corresponds to 5 and 95 percentiles. Elongation and area data are presented as mean ± SD. ns = not significant, *p < 0.01; one-way analysis of variance (ANOVA).

To investigate the strain-dependent response of the cytoplasm and nucleus to dynamic stretches, we applied uniaxial cyclic stretches of 5%, 10%, and 15% strains at 0.3 Hz to A549 cells cultured on a PDMS well for 15, 30, 60, and 120 min. After the 2-h-long cyclic stretch, the mechanical stretch to the PDMS well was released. The morphological features of the cytoplasm and nucleus, including their orientation angles, elongation factors, and area, were measured or calculated at each time point both in the stretch and release modes (Fig. 2A–C). Unstretched cells (i.e. cells at 0 min) showed a broad distribution of orientation angles of the cytoplasm and nucleus with the median angle of 60° and 58°, respectively. The cytoplasm and nucleus of the stretched cells were preferentially reoriented in an oblique angle to the stretch direction (Fig. 2A). The 2 h-long cyclic stretches resulted in a narrow range of the orientation angle of the cytoplasm with median angle of 76° regardless of strain magnitudes (Fig. 2B). The orientation angles (in median values) of the nucleus at 120 min were 68°, 73°, 75° at 5%, 10%, 15% strains, respectively (Fig. 2C).

Interestingly, we could see the larger difference in the orientation angle between the cytoplasm and nucleus at 5% (i.e. 8°) than at 10% (i.e. 3°) or 15% (1°), indicating that the nucleus showed relatively a slow response compared to the cytoplasm at a small strain while a large strain made their orientation angles almost identical within 120 min. To quantify the kinetics of reorientation angles of the cytoplasm and nucleus, time constants were extracted from their reorientation profiles that were fit to exponential curves (Table 1 and Supplementary Fig. S6A). As expected, the lager strains led to faster reorientation of the cytoplasm and nucleus of A549 cells. The time constants of the cytoplasm and nucleus under 5% strain were 58.7 and 99.2 min, respectively, whereas those of cytoplasm and nucleus under 15% strain were 10.5 and 9.5 min, respectively. The large difference in time constants between the cytoplasm and nucleus at 5% strain supported the lagged reorientation of the nucleus while small and similar time constants at 15% strain favored the rapid and concurrent reorientation of the cytoplasm and nucleus. Additionally, a large strain caused more elongated shapes of cells and faster changes in cytoplasmic and nuclear morphology than a small strain (Supplementary Table S1). Cells that were stretched at 15% strain showed a 1.4-fold increase in the elongation parameter at large strains (10% and 15%) in comparison to unstretched or 5% stretched cells. However, no significant change in the area of the cytoplasm and nucleus was observed (Fig. 2B,C).

**Table 1:** Time constant values of reorientation and recovery in epithelial cells under cyclic stretch (CS) and post-CS release.

Strain (%)	Cyclic stretch (CS)		Post-CS release	
	Cell body	Nucleus	Cell body	Nucleus
5	58.7 min	99.2 min	36.9 min	NA^a^
10	22.2 min	30.6 min	72.7 min	NA
15	10.5 min	9.5 min	87.8 min	NA

^a^NA (not available): the exponential model does not fit to the kinetic profile.

To determine whether the release of cells from cyclic stretches would lead to the recovery of the cytoplasm and nucleus, and whether their recovery kinetics would be affected by the strain magnitude, we performed time-lapse imaging of the cells after the 2 h-long stretches. Cells that were cyclically stretched at 5% recovered the broad range of orientation angles as observed in unstretched cells while those that were stretched at 10% and 15% showed their partial recovery for 2 h (Fig. 2A–C). When time constants in the release phase were compared, cells that underwent large strains showed the slower recovery of the cytoplasm orientation than those exposed to a small strain (Table 1). Because the release kinetic profiles for the nucleus orientation were not fit to the exponential curve, those time constants could not be extracted. Along with the orientation recovery of cells, it seemed that their stretch-induced elliptical shapes eventually could return to the initially unstretched shape. The elongated cytoplasm recovered its initial shape within 2 h regardless of the strain magnitude whereas the nucleus remained slightly elongated at 2 h since the release of 10% or 15% strain, suggesting the relatively slower morphological recovery of the nucleus compared to the cytoplasm.

### Stress fiber subtypes were reversibly reorganized in the presence or absence of cyclic stretches

**Figure 3:**
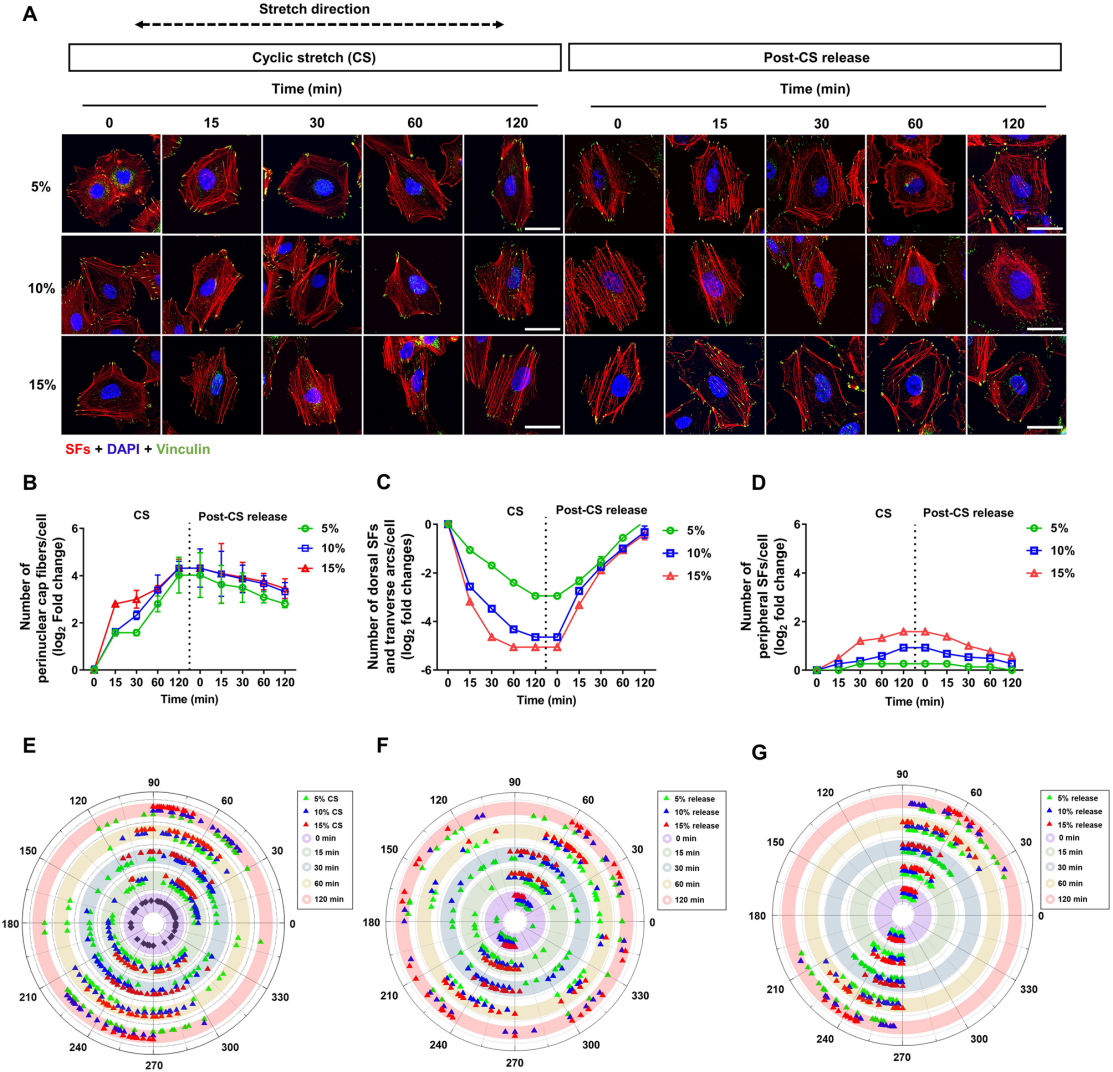
Reorganization of stress fibers (SFs) subtypes during uniaxial cyclic stretch (CS) and post-CS release. (**A**) Cells were stained with rhodamine-phalloidin (SFs), DAPI (nucleus), and anti-vinculin antibody (vinculin) after stretching (upper panel) and post-CS release (lower panel). Scale bars: 100 µm. Quantitative analysis of the number of (**B**) perinuclear cap fibers, (**C**) transverse arcs and dorsal SF, (**D**) peripheral SFs and perinuclear SFs in cells subjected to CS and post-CS release (n = 30). Error bars indicate mean ± SD. (**E**) The remodeling of focal adhesion complexes (FACes) in stretched cells was presented by polar plot (n = 5). (**F**) The recovery of FACes distribution after the release of CS (n = 5). (**G**) The recovery of FACes associated with perinuclear cap fibers after the release of CS (n = 5).

To understand how SF subtypes are involved in strain-dependent reorientation of the cytoplasm and nucleus to uniaxial cyclic stretches, we examined the reorganization of SF subtypes through the fluorescence staining of SF-associated molecules. The cells were immediately fixed and stained for actin filaments and FAC molecules (vinculin) after applying cyclic stretches to cells. Z-stack images were obtained using a confocal fluorescence microscope to identify SF subtypes. The cyclic stretches significantly altered the organization of SF subtypes in A549 cells (Fig. 3A). In most unstretched cells, the typical localization of dorsal SFs, transverse arcs, and peripheral SFs were observed; however, few perinuclear cap fibers were detected (Fig. 3A and Supplementary Fig. S5). The cyclic stretch-induced noticeable changes in the number and distribution of SF subtypes. The number of perinuclear cap fibers in stretched cells increased by 16-to-20-fold (4.0-to-4.3 in log_2_ fold change) relative to that found in unstretched cells whereas the number of dorsal SFs and transverse arcs was strain-dependently deceased at 2 h of cyclic stretches (Fig. 3B,C): approximately 8-fold decrease at 5% strain and 24-fold decrease at 10% or 15% strains. The number of peripheral SFs slightly increased at 10% or 15% strains, but no significant change in their numbers at 5% (Fig. 3D). In addition, the length of peripheral SFs showed a similar pattern of change: 1.3- to 1.5-fold at 10% or 15% strains but insignificant changes at 5% strain (Supplementary Fig. S6B).

We also observed the recovery of SF subtypes after the release of cyclic stretches. They exhibited different rates and levels of the recovery in their cytoplasmic distribution. A considerable number of perinuclear cap fibers were observed in the apical region of the nucleus with gradual decrease in their number over the relaxation phase: at 2 h of release, 8- to 10-fold higher number compared to that counted in initially unstretched cells (Fig. 3B). Dorsal SFs and transverse arcs rapidly reappeared and their numbers recovered to ca. 100%, 80%, and 75% within 2 h in cells that were released from 5%, 10%, and 15% strains, respectively (Fig. 3C). The number of peripheral SFs gradually decreased after releasing from 10 and 15% strains, reaching its initial level within 2 h (Fig. 3D).

The circumferential distribution focal adhesion complexes (FACes) around individual cells were represented on a circular chart (Fig. 3E). Unlike the relatively uniform FAC distribution in unstretched cells, the strain-dependent relocalization or reorientation of FAC was observed: the large strain gave rise to the more dominant formation of FAC clusters at both ends of the major axis of elongated cells with the significant loss of lateral FACes compared to the small strain. After 2 h of cyclic stretches, the cluster angles for polar FACes in 5%, 10%, and 15%-strained cells were approximately 60°, 40°, and 10°, respectively. Most FACes were clustered more narrowly as a given strain was applied for a longer time. After cyclic stretch release, the recovery of FACs orientation has been observed (Fig. 3F). However, those associated with perinuclear cap fibers were maintained in clusters (Fig. 3G).

### Redistribution of myosin II in stress fibers in response to cyclic stretches

**Figure 4:**
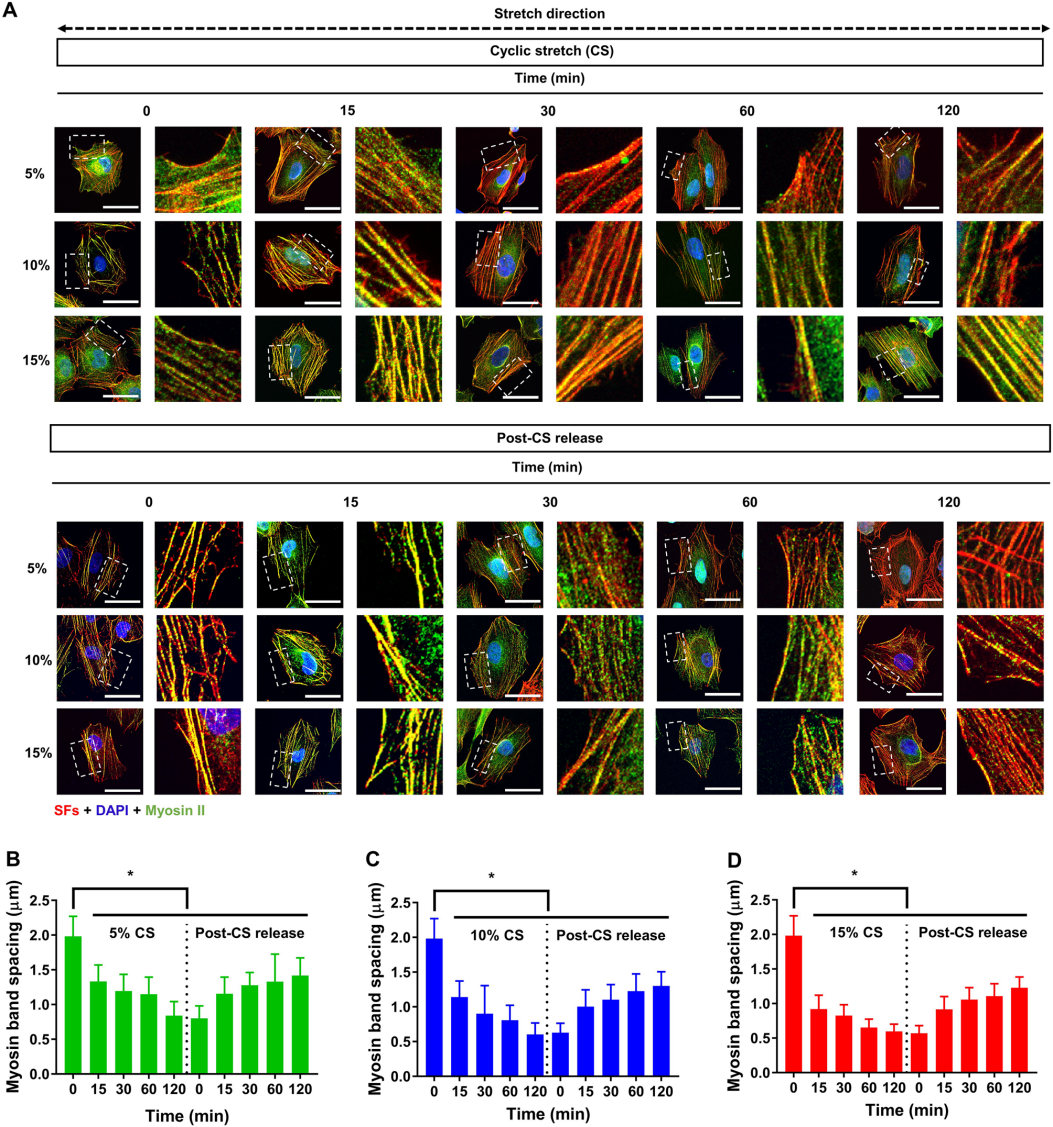
Dynamic redistribution of myosin II in stress fibers (SFs). (**A**) Distinct distribution of myosin II in unstretched cells and after applying cyclic stretch (CS) for 2 h (upper panel). Redistribution of myosin II post-CS release (lower panel). Scale bars: 100 µm. (**B–D**) Remodeling of myosin II is quantified by the spacing between myosin bands along with SFs in cyclically stretched cells at 5% (**B**), 10% (**C**), and 15% (**D**) strains (n = 3). Error bars indicate mean ± SD. *p < 0.01; one-way analysis of variance (ANOVA).

It has been reported that the contractile peripheral SFs, transverse arcs, and perinuclear cap fibers show periodic bands of myosin II that are incorporated into them in contrast to its rare and irregular distribution in non-contractile dorsal SF^12,14^. We investigated whether myosin II remodeling is involved in the reorganized SF subtypes such as peripheral SFs and perinuclear cap fibers during the cyclic stretches and subsequent stretch release. The confocal microphotographs showed a distinct distribution of myosin II bands along with peripheral SFs in stretched cells whereas myosin II bands were not clearly observed along perinuclear cap fibers which were relatively thin in unstretched cells (i.e. at 0 min) (Fig. 4A). Unstretched cells showed a random distribution of myosin II bands. When cells were subjected to cyclic stretches with three different strains myosin II bands were observed to be redistributed along the peripheral stress fibers and perinuclear cap fibers within 15 min, suggesting rapid incorporation of myosin II into these SFs in response to cyclic stretches. When released from the cyclic stretch, scattered myosin II bands were observed inside cells after 2 h. Since the periodic myosin II band is a typical signature for the contractility of peripheral SFs^12,21^, we examined the extent of myosin II remodeling in stretched cells by measuring the microscale distance between neighboring myosin II bands along with peripheral SFs (Fig. 4B–D). The average of myosin band II spacing in unstretched cells was 1.9 ± 0.3 µm. However, the application of cyclic stretches of 5%, 10%, and 15% strains to these cells for 2 h significantly decreased the myosin band II spacing in a strain-dependent manner to 0.8 ± 0.4 µm, 0.6 ± 0.3 µm, and 0.5 ± 0.2 µm, respectively. The stretch release led to the partial (65% to 70%) recovery of myosin band II spacing after 2 h: 1.41 ± 0.25 µm, 1.3 ± 0.2 µm, 1.21 ± 0.1 µm released from 5%, 10%, and 15% strains, respectively.

### Blebbistatin inhibits stretch-induced stress fibers reorganization

**Figure 5:**
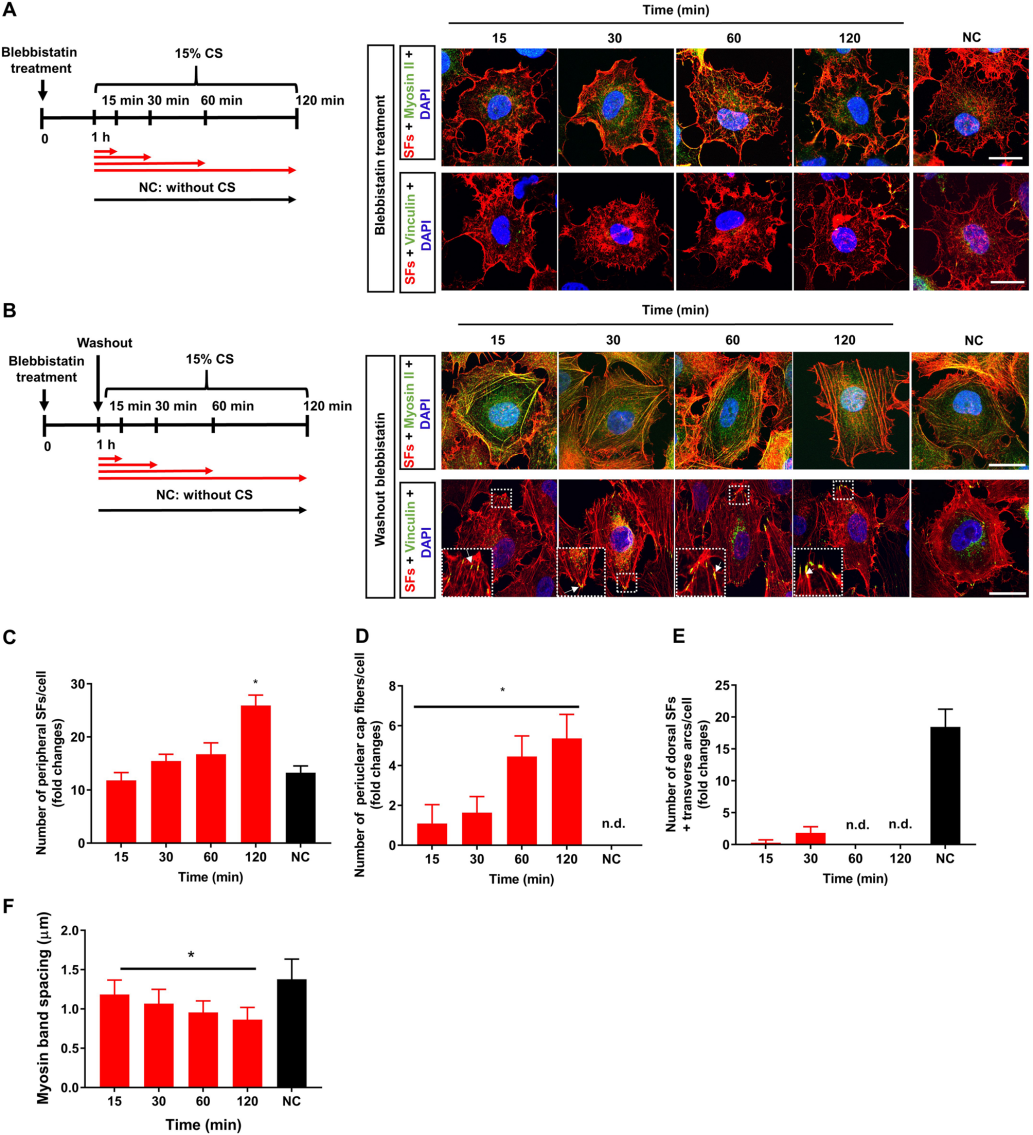
Effect of blebbistatin on stress fibers (SFs) remodeling and cell reorientation. (**A**) Prior to cyclic stretch (CS), cells were treated with 50 µM blebbistatin for 1 h and exposed to 15% strain in the presence of blebbistatin for the indicated time points. Cells were stained with rhodamine-phalloidin (SFs) and DAPI (nucleus) anti-myosin antibody (myosin) and anti-vinculin antibody (vinculin) after stretching. Negative control (NC) cells were treated with blebbistatin and immediately fixed at 3 h endpoint. Scale bars: 100 µm. (**B**) Prior to CS, cells were treated with 50 µM blebbistatin for 1 h, washed thrice with phosphate-buffered saline (PBS), and subjected to 15% CS in the absence of blebbistatin. Cells were stained with rhodamine-phalloidin (SFs) and DAPI (nucleus); myosin and vinculin after stretching. Scale bars: 100 µm. Quantitative analysis of the number of (**C**) peripheral SFs, (**D**) perinuclear cap fibers, (**E**) dorsal SFs, and transverse arcs (n = 30). (**F**) Myosin band spacing after applying CS in the absence of blebbistatin (n = 3). Error bars indicate mean ± SD. *n.d. *not detected *p < 0.01; one-way analysis of variance (ANOVA).

To confirm the association of myosin II remodeling with the reorganization of SF subtypes, we pharmacologically perturbed SF contractility with blebbistatin that has been used as an inhibitor of myosin II ATPase activity in cells^23^. In this study, blebbistatin was applied further to investigate the reformation of SF subtypes under cyclic stretch. The range of 50–100 μM of blebbistatin has been previously reported as a nontoxic concentration for the cells^12,21,24^ and we confirmed no significant change in cell viability at 50 μM (Supplementary Fig. S4C,D). As illustrated in Fig. 5A, epithelial cells were pretreated with 50 μM blebbistatin for 1 h and exposed to 15% cyclic stretches for the indicated time points in the presence of blebbistatin. Cells were immediately fixed and stained for the detection of actin filaments, myosin II, and vinculin. Blebbistatin prevented myosin II from incorporating into actin filaments leading to the disappearance of SFs and the formation of star-shaped networks of actin filaments along the cytoplasm membrane (Fig. 5A). The vinculin spots for FACes were sparsely observed when myosin II was inhibited. The subsequent application of cyclic stretches to blebbistatin-treated cells induced no noticeable changes in cell orientation and morphology as well as no SF configuration and FAC regain within 2 h. The reversibility of the configuration of SF subtypes was also investigated after washed out of blebbistatin from cells followed by 15% cyclic stretches (Fig. 5B). Blebbistatin-treated but unstretched cells (i.e. NC) recovered typical networks of SF subtypes such as peripheral SFs, dorsal SFs, and transverse arcs except for perinuclear cap fibers after 2 h of blebbistatin wash-out. When the cyclic stretch was applied to cells that had been removed from blebbistatin, the significant increase in the number of peripheral stress fibers and perinuclear cap fibers were observed (Fig. 5C,D). The number of peripheral stress fibers increased by 2.3-fold after 2 h compared to unstretched cells. The cyclic stretch resulted in the recovery of perinuclear cap fibers in 15 min and ca. 5-fold increase in their number in the next 105 min. Dorsal stress fibers and transverse arcs showed a slight increase in their numbers in the early phase and then disappeared afterward (Fig. 5E). Moreover, as a result of the cyclic stretch, the spacing of myosin II bands gradually decreased to 0.8 ± 1 µm (Fig. 5F).

## Discussion

**Figure 6:**
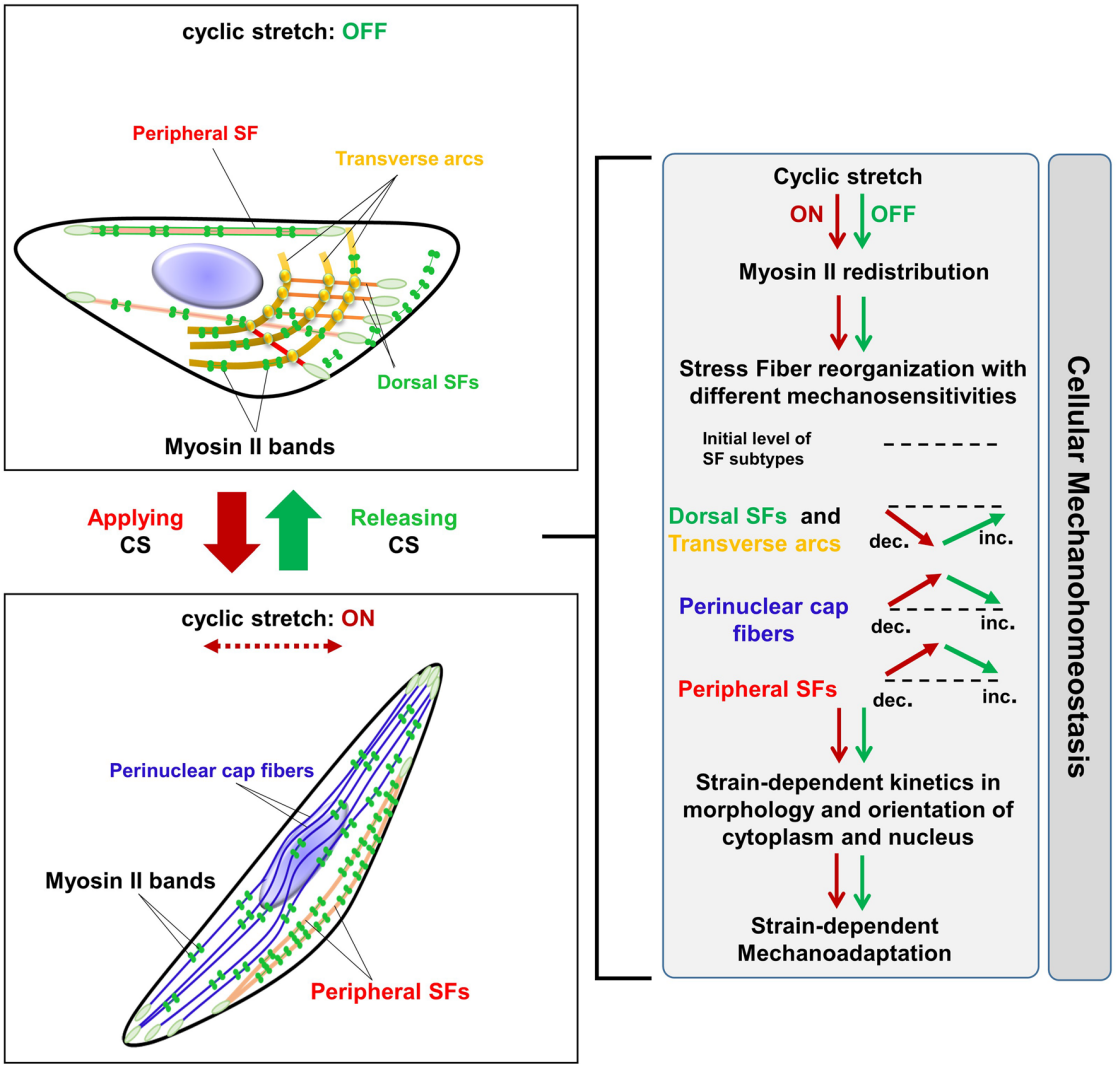
Working model for the structural adaptation of epithelial cells in response to cyclic stretch (CS). Schematic drawing of the effects of uniaxial cyclic strain on stress fiber (SF) orientation of epithelial cells cultured on polydimethylsiloxane (PDMS) aligned with the direction of strain. Alveolar epithelial cells subjected to uniaxial CS at three different strains (5%, 10%, and 15%) undergo morphological alteration and reorientation. These mechanical stimuli induce elliptical shapes of the cell bodies and nuclei that are aligned in an oblique angle to the stretch direction with strain-dependent kinetics. In the presence of CS, the number of peripheral SFs and perinuclear cap fibers are observed to be increasingly prominent in, while that of dorsal SFs and transverse arcs are continuously decreased. In additions, dorsal SF and transverse arcs are more mechanosensitive to CS than other SF subtypes. Dorsal SFs, transverse arcs, and peripheral SFs are involved in the fast response of cell body whereas perinuclear cap fibers are mainly closely related to slower kinetics of the nuclear responses. Through their interconnection, the reorganization of SFs resulted in strain-dependent mechanoadpative in cyclically stretched epithelial cells. The organization of SF subtypes occurred in accordance with the remodeling of myosin II in uniaxially stretched cells. The reversible distribution of myosin II and SFs was induced post-CS release that led to the recovery of morphology and orientation of cells to their initial state.

On the basis of our findings, we propose a model in which uniaxial cyclic stretches induce the strain-dependent responses of the cytoplasm and nucleus of epithelial cells through mechanoadaptive reorganization of SF subtypes (Fig. 6). Upon cyclic stretches, peripheral SFs and perinuclear cap fibers become increasingly prominent and obliquely reoriented with respect to the stretch direction, resulting in the inclined reorientation and the elongated morphology of cells. The number of dorsal SFs and transverse arcs rapidly decreases in response to the cyclic stretch. On the other way, when cells are released from cyclic stretches, the increased number of perinuclear cap fibers and peripheral SFs is partially or completely reduced to their original numbers determined in initially unstretched cells. Relatively rapid recovery of dorsal SFs and transverse arcs occur in stretch-released cells. The recovery of the configuration of SF subtypes restores the cell orientation and morphology observed in initially unstretched cells. Additionally, myosin II reversibly mediates the reorganization or remodeling of SF subtypes in cells subjected to the cyclic stretch or stretch relaxation.

The reorganization of dorsal SFs, transverse arcs, and peripheral SFs were closely correlated to the reorientation and elongation of the cytoplasm under both cyclic stretches and post-stretch release. We found that dorsal SFs and transverse arcs were the most mechanosensitive among SF subtypes since they were rapidly disassembled under mechanical stretches. Within the first 15 min, the significant decrease in the number of dorsal and transverse arcs was observed when cells were subjected to cyclic stretches (Fig. 3C). It has been reported that dorsal SFs and transverse arcs play an important role in maintaining the cell shape^13^. Therefore, the initial decrease of dorsal SFs may facilitate the subsequent reorientation and elongation of the cytoplasm. As some dorsal SFs and transverse arcs could be converted to peripheral SFs^12^, the conversion might lead to increase in the number of peripheral SFs by 1.9-and-3-fold after 2 h in response to 10%, and 15% strains, respectively (Fig. 3D). We also assumed that the rapid decrease in number of dorsal SFs and transverse arcs also could enable the inclined orientation (Fig. 3A) as well as increase in the elongation of peripheral SF. Subsequently, the inclined alignment of peripheral SFs resulted in the reorientation of the cytoplasm at 76° after 2 h regardless of strain magnitudes (Fig. 2A). The final angle of reorientation of the cytoplasm was determined by combined effect of tensile and compressive strains^2,17^, which can be theoretically represented by the identical biaxial ratio (− ε_yy_/ε_xx_), 0.49, at the central region of PDMS well at the three strains (Supplementary Fig. S1C). In addition, the 1.3- to 1.5-fold increases in the length of peripheral SFs were fit to the 1.4-fold increase in the cell elongation parameter at large strains (Fig. 2B). Noticeably, dorsal SFs and transverse arcs were also recovered rapidly upon the release of cyclic stretches. The assembly of dorsal SFs and transverse arcs increased 3.8- to 4.0-fold (in linear scale) when cells were released from the large strains after 15 min (Fig. 3C). This suggested that the rapid assembly of dorsal SFs and transverse arcs might be necessary for the return of the elongated and reoriented cytoplasm to their initial shapes and orientations. Taken together, these data clarified that rapid responses of dorsal SFs and transverses arcs are necessary for the reorganization of peripheral SFs, which subsequently induces the morphological alterations of the cytoplasm under cyclic stretches or post-stretch release.

Perinuclear cap fibers were mainly involved in nuclear reorientation and elongation in response to mechanical cues. Unlike other SFs, perinuclear cap fibers could sense the mechanical tension in extracellular matrix through their linking to FACes and subsequently regulate the nucleus response through LINC (linker of nucleoskeleton and cytoskeleton) complex^7–9^. Thus, these fibers and LINC complex are the major structures in the regulation of nuclear shape, reorientation, and nuclear anchoring^5,7,10^. In this work, we observed the strain-dependent reorientation of the cytoplasm and the nucleus in response to cyclic stretches. Large strains induced the concurrent reorientation of the cytoplasm and nucleus. The time constant of the cytoplasm and nucleus were 10.5 and 9.5 min, respectively, at 15% strain (Table 1). However, the nucleus showed a lagged kinetics of stretch-induced reorientation compared to the cytoplasm at small strain (Fig. 2B,C). This may attribute to the slow formation of perinuclear cap fibers at the small strain in comparison to their formation at the large strain. Remarkably, we observed the partial recovery of the nucleus orientation and morphology after 2 h of stretch release (Fig. 2C). Furthermore, perinuclear cap fibers were not completely disassembled when cells were released from the cyclic stretches. The number of perinuclear cap fibers was maintained 2-to threefold higher compared to unstretched cells (Fig. 3B). The FACes that were connected to perinuclear cap fibers were maintained in the cluster form after the release of cyclic stretches (Fig. 3G). These results suggested that the slow recovery of the nucleus orientation and morphology might be contributed to the slow response of perinuclear cap fibers. Collectively, the formation and stability of perinuclear cap fibers were correlated to the strain-dependent kinetics of nuclear reorientation and elongation in both the presence and the absence of cyclic stretches.

The SF subtype-specific incorporation pattern of myosin II might be responsible for remodeling SF subtypes reorganization. Myosin II is a main component of SFs that coordinate the contractile property of peripheral SFs, and transverse arcs^12,14^. Blebbistatin treatment prevented the incorporation of myosin II into SFs resulting in dramatic disappearance of SFs in epithelial cells and loss of stretch-induced responses of the cytoplasm and nucleus (Fig. 5A). This confirmed that myosin II remodeling was a major player in the reorganization of SF subtypes in response to cyclic stretches. When we analyzed the kinetic profile of the redistribution of myosin II from lamellipodia into peripheral SFs that occurred almost at the same time as the disassembly of dorsal SFs; the time constant values were 10.5 and 7.8 min at 15%, respectively (Supplementary Table S2 and Supplementary Fig. S6B). Along with the disassembly of dorsal SFs, their associated FACes at the lateral side of the cytoplasm was disappeared (Fig. 3E). Therefore, we proposed that the retrograde flow of myosin II could inhibit the maturation of FAs at lamellipodia that subsequently led to the inhibition of dorsal SFs polymerization in cells. This hypothesis was consistent with the findings of previous studies^24,25^ in which the maturation of FACes was shown to be dependent on the activation of myosin II. In addition, some exiting dorsal SFs and transverse arcs could be converted to peripheral SFs and perinuclear cap fibers^10,26^. The subsequent formation of peripheral SF and perinuclear cap fibers were associated with the forming of FACes cluster in stretched cells (Fig. 3E). The increase in peripheral SF and perinuclear cap fibers subsequently induced the reorientation of the cytoplasm and nucleus with the time constants: 10.5 and 9.5 min at 15% strain (Table 1). The partial disappearance of dorsal SFs and transverse arcs caused by F-actin stabilizing agent, Cucurbitacin E (CuE) resulted in the slow transition of SFs (i.e. dorsal and transvers arcs) to perinucleus SFs and the subsequent retardation of the cellular reorientation in response to 15% CS (Supplementary Fig. S7). It has been shown that applying 10% equibiaxial cyclic stretch-induced the 1.5-fold increase in phosphorylation of FAK (Y397) in pulmonary epithelial cells at the early 15 min^27^. In this work, to verify whether the uniaxial cyclic stretch could upregulate the expression level of mechanosensitive signature proteins in A549 cells, we performed western blotting for p-FAK (Y397) and p-Paxillin (Y118) in cells that were subjected to 15% cyclic stretch for 2 h. However, our results showed no significant changes in the total and phosphorylated levels of paxillin when cells were applied to cyclic stretches for 15 min or 120 min. Noticeably, we detected 1.6-fold increase in p-FAK expression when cells were subjected to cyclic stretch for 15 min compared to cells at 0 min, suggesting that the phosphorylation of FAK might have a critical role in mechanosensing at the early stage but not late stage to adapt cyclic stretches (Supplementary Fig. S8). Altogether, these findings suggested that the incorporation of myosin II to peripheral SFs could lead to the dynamic organization of dorsal SFs, transverse arcs, peripheral SFs, and perinuclear cap fibers in cyclically stretched cells. The reorganization of SF subtypes subsequently induced the strain-dependent responses of the cytoplasm and nucleus. Future studies are necessary to clarify the molecular mechanism that regulates different mechanosensitivity of SF subtypes and the crosstalk between myosin II and remodeling FAC components like FAK, paxillin, zyxin or talin during the reorganization of SF subtypes in response to mechanical cues.

In conclusion, our findings demonstrated the kinetically linked coordination of the reorganization of SF subtypes in the stretch-induced response of the cytoplasm and nucleus of epithelial cells. Dorsal SFs and transverse arcs exhibited high mechanosensitivity compared to peripheral SFs in the reorientation and elongation of the cytoplasm under cyclic stretches. In addition, the slow formation of perinuclear cap fibers was associated with the lagged kinetics of nuclear reorientation at small strain, while high stability contributed to the partial recovery in nuclear orientation and morphology after cyclic stretches release. The reorganization of stress fiber subtypes was driven by switching the distribution of myosin II rapidly and reversibly to adjust their contractility, maintaining cellular mechanohomeostasis in response to mechanical stretches or strain release.

## Supplementary information

Supplementary Information.
