# Supplementary material for: Mechanoadaptive organization of stress fiber subtypes in epithelial cells under cyclic stretches and stretch release
Source: Sci Rep. 2020 Oct 29;10:18684. doi: 10.1038/s41598-020-75791-2 (PMC7596055; doi:10.1038/s41598-020-75791-2)
Supplement: Supplementary file 1 — Supplementary Information. [file 41598_2020_75791_MOESM1_ESM.pdf]

Supplementary Information

Mechanoadaptive Organization of Stress Fiber Subtypes in Epithelial Cells under Cyclic Stretches and Stretch Release

Amir Roshanzadeh[1,§], Nguyen Thi Tham[1,§], Nguyen Dang Khoa[1,§], Dong-Su Kim[2], Bong-Kee Lee[2,4], Dong-Weon Lee[2,4], Eung-Sam Kim[1,3,4,*]

[1]School of Biological Sciences and Biotechnology, Chonnam National University, Gwangju, Republic of Korea, [2]Department of Mechanical Engineering, Chonnam National University, Gwangju, Republic of Korea, [3]Department of Biological Sciences and Research Center of Ecomimetics, Chonnam National University, Gwangju, Republic of Korea, [4]Center for Next Generation Sensor Research and Development, Chonnam National University, Gwangju, Republic of Korea.

§ These authors contributed equally to this work.

*** Correspondence: Eung-Sam Kim, Ph.D.**

Department of Biological Sciences and Research Center of Ecomimetics, Chonnam National University, Gwangju, 61186, Republic of Korea, E-mail: eungsam.kim@chonnam.ac.kr, Tel: +82-62-530-3416; Fax: +82-62-530-3409

Table S1. Time constant value of elongation and recovery in alveolar epithelial cells under cyclic stretch (CS) and post-CS release.

Strain (%)	Cyclic stretch (CS)		Post-CS release	
	Cell Body	Nucleus	Cell Body	Nucleus
**5**	NA*	NA	NA	NA
**10**	35.3 min	44.8 min	60.4 min	71.7 min
**15**	16.6 min	20.1 min	82.6 min	47.2 min

* NA (not available): the exponential model does not fit to the kinetic profile; small strain shows no discernible effect to elongation of the cell body and nucleus.

Table S2. Time constant value of myosin II remodeling in cells under cyclic stretch (CS) and post-CS release.

Strain (%)	Cyclic stretch (CS)	Post-CS release
**5**	NA	NA
**10**	NA	NA
**15**	10.5 min	14.0 min

Figure S1. Polydimethylsiloxane (PDMS) wells, strain profiles and finite element model linear analysis of PDMS membrane deformation under 5%, 10%, and 15% strains. (A) Design of a metal mold and fabrication of PDMS wells [inner dimensions of a single PDMS well: 4 cm (length) × 2.4 cm (width) × 0.3 cm (height); outer dimensions: 5 cm × 3 cm × 0.35 cm]. Each PDMS cell well was fabricated through the assembly of a side wall and a flat bottom, both made of PDMS, through O_2 plasma treatment. **(B)** Strain profiles applied to A549 cells. ACC: acceleration time from motor speed; DCC: deceleration time until the motor speed reaches range, 1–9999 ms. +Pause: pause time before starting to stretch; - Pause: pause time during the stretched condition. **(C)** The uniformity of PDMS deformation was analyzed using finite element model. PDMS membrane showed the same biaxial ratio ($-\varepsilon_{yy}/\varepsilon_{xx} = 0.49$) at the central region (1 mm × 1 mm) for 5%, 10% and 15 % strains.

Figure S2. The image-processing procedure to define the boundary of individual cells in phase contrast images. (Step 1) phase contrast images were imported to ImageJ, (Step 2) the contrast and brightness were adjusted, and (Step 3) magnified images of cellular boundaries (indicated with red lines).

Figure S3. Distribution of myosin II bands along the stress fibers. The myosin II band spacing was determined by averaging the center-to-center linear distance between two neighboring myosin II bands in the enlarged fluorescence image (GFP: myosin II).

Figure S4. Effect of cyclic stretch (CS) on cell viability. (A) Cell viability of A549 cells

measured through the Live/Dead assay after cyclic stretched at 5%, 10%, 15% strains, and 15% strain with 50 μM of blebbistatin. Scale bars: 100 μm. **(B)** The quantification of dead cell to total cell number showed no significant difference at 5% and 10%, 15% strain, and 15% with blebbistatin. **(C)** Effect of blebbistatin treatment on A549 cells viability after 1 h. **(D)** The quantification of dead cells to total cell number when cells were treated with 20 μM, 40 μM, and 80 μM of blebbistatin.

Figure S5. Z-stack images of A549 under unstretched and stretched conditions at 15% strain for 2 h. The distance for each layer was 0.4 μm, and the laser scanning was performed from the bottom (Z1) to top (Z12).

Figure S6. Exponential fitting model for the response of the cell body, nucleus, dorsal stress fibers, and myosin II. **(A)** Cell and nucleus reorientation and elongation showed the characteristics of an exponential increase between limits, while the recovery process was fitted by an exponential decrease between limits. **(B)** Dorsal SFs disassembly fitting model and changes in the lengths of dorsal SFs and peripheral SFs at 5%, 10%, and 15% strains. **(C)** Myosin II remodeling under uniaxial cyclic stretch (CS) and recovery of myosin II in post-CS release.

Figure S7. Effects of Cucurbitacin E (CuE) on cell viability and actin filament distribution in A549 cells. (A) Cells pretreated with various concentrations of CuE for 2 h and then cell viability was evaluated by WST assay. **(B)** Cell viability of A549 cells measured

through the Live/Dead assay after 2 h treatment of CuE at different doses. Scale bars: 100 μm. **(C)** Actin filaments were stained with phalloidin after treatment of CuE at 10 and 20 nM under unstretched condition. Scale bars: 30 μm. **(D)** Actin filaments were stained with phalloidin after treatment of CuE at different time points under 15% CS. Scale bars: 30 μm. **(E)** The kinetic profile of cell reorientation under 15% CS without or with the treatment of CuE was presented in the box–whisker plot (n = 50).

Figure. S8. Western blots of mechanosensitive signature proteins in A549 cells under 15% uniaxial cyclic stretch. (A) Scheme for membrane cuts and **w**estern blot images of target protein expression in three different membranes with consideration of the molecular weight; Membrane 1 was used for detection of p-FAK (Tyr397), β-actin, and stripped T-FAK; Membrane 2 for T-Myosin IIa and p-Paxillin (Tyr118); Membrane 3 for detection of T-paxillin. **(B)** Western blot images were presented in the upper side with the indicated areas of the selection in the blots with **(C)** 2 s- and **(D)** 5 s-long exposure. **(E)** Replicate western blot images of target protein expression with the indicated areas of the selection in the blots were shown in the right side. The protein name and size marker were indicated in the left and right side of each blot, respectively.

Figure S1

A

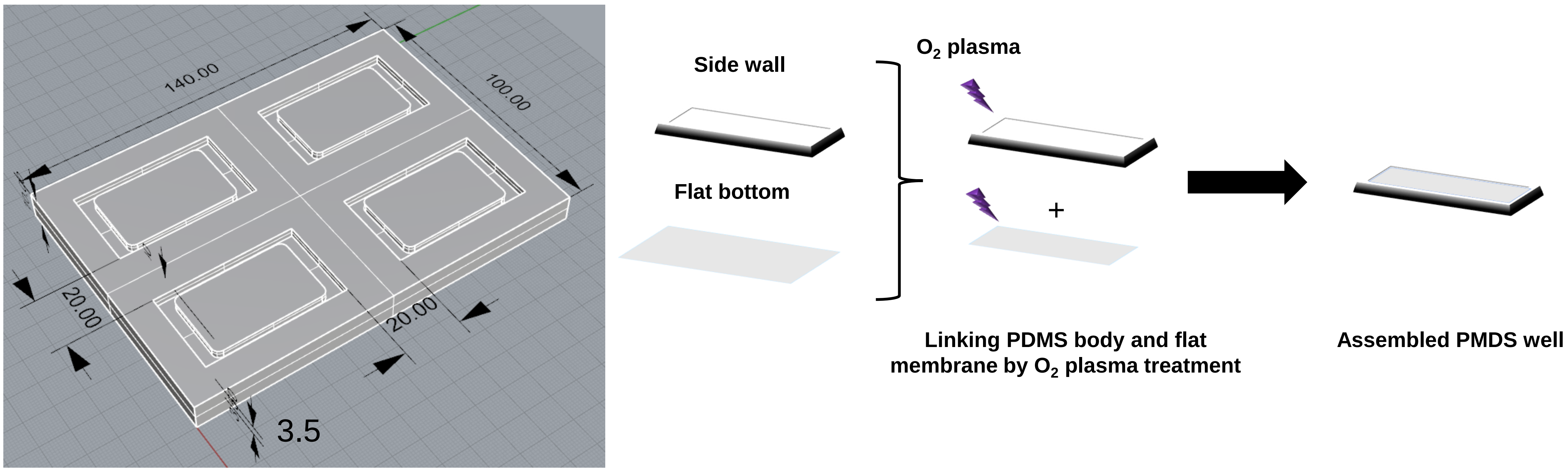

B

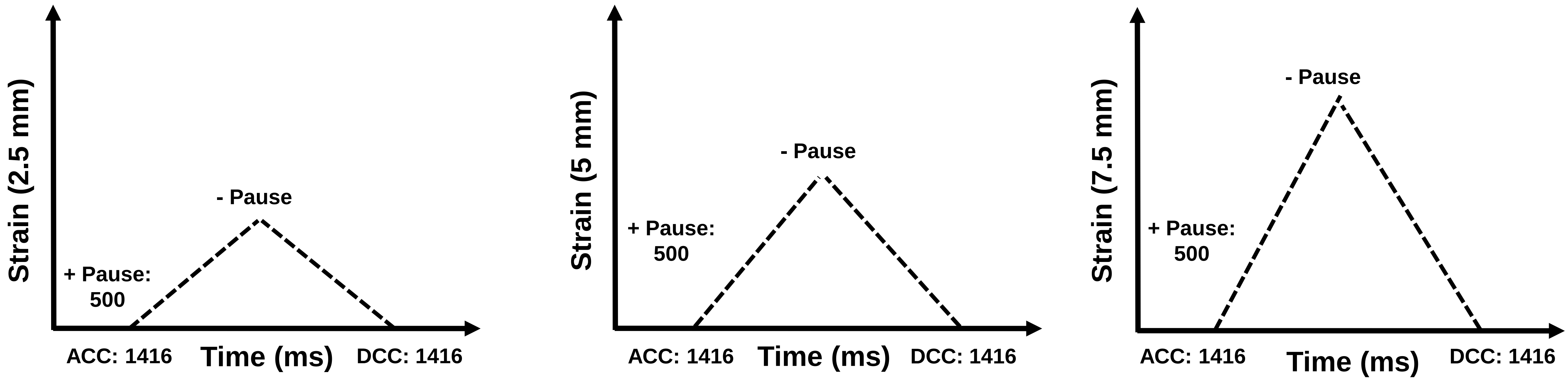

C

Strain	5%	10%	15%
Surface: von Misses stress (N/m²)	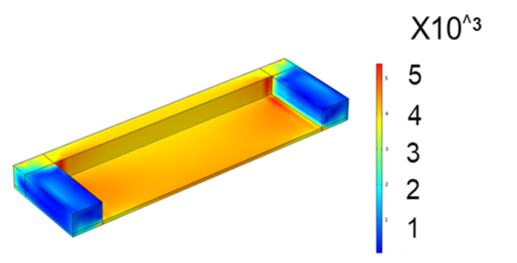	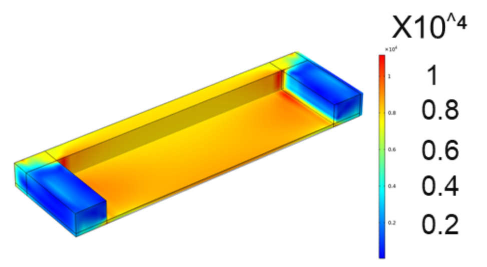	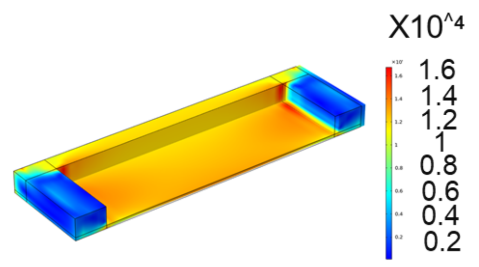
Strain in x direction (ε_{xx})	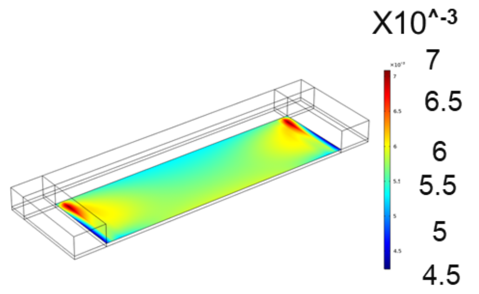	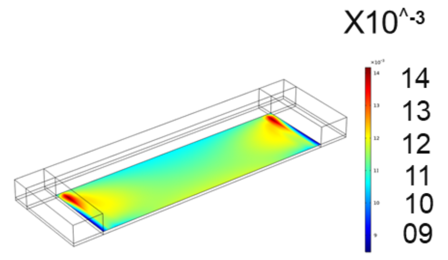	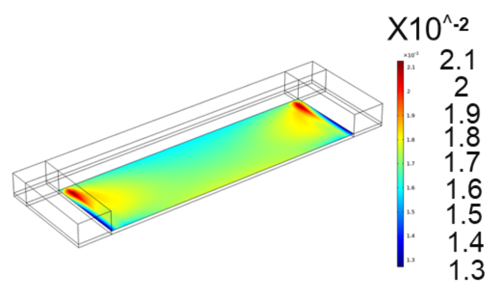
Strain in y direction (ε_{yy})	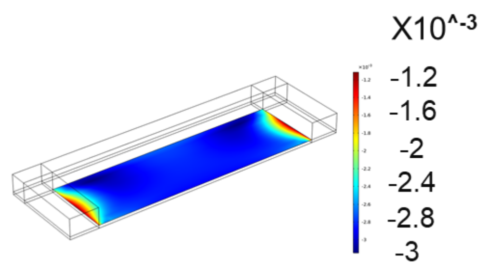	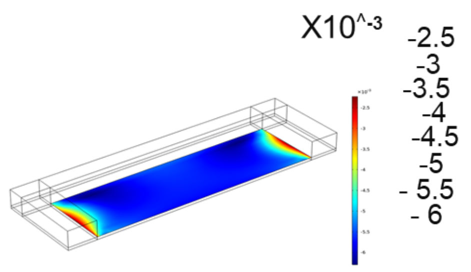	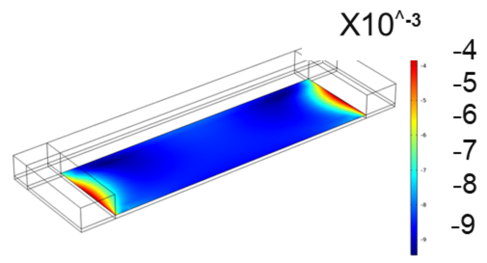
Biaxial ratio ($-\varepsilon_{yy}/\varepsilon_{xx}$)	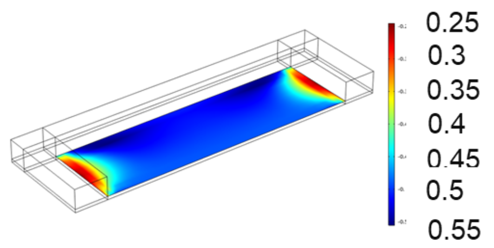	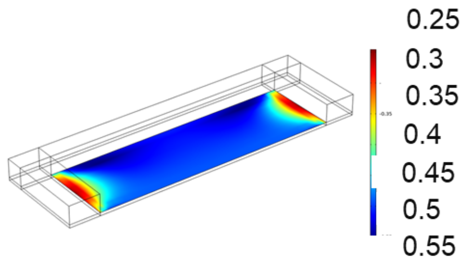	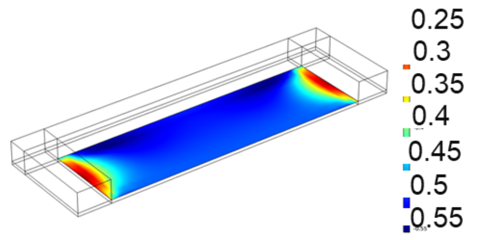

Figure S2

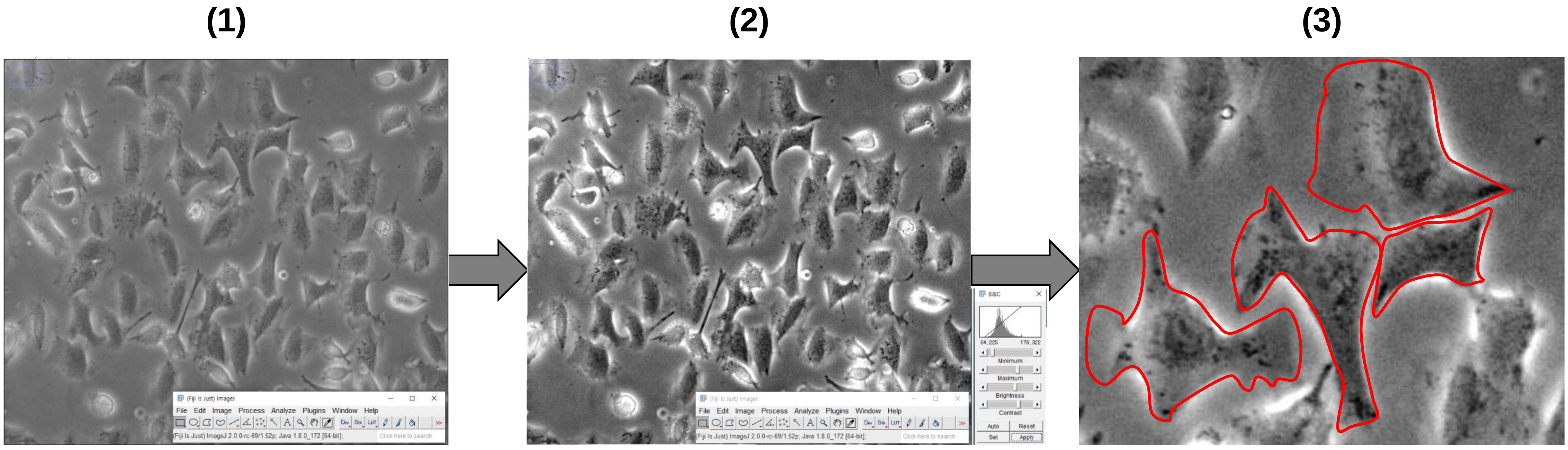

Figure S3

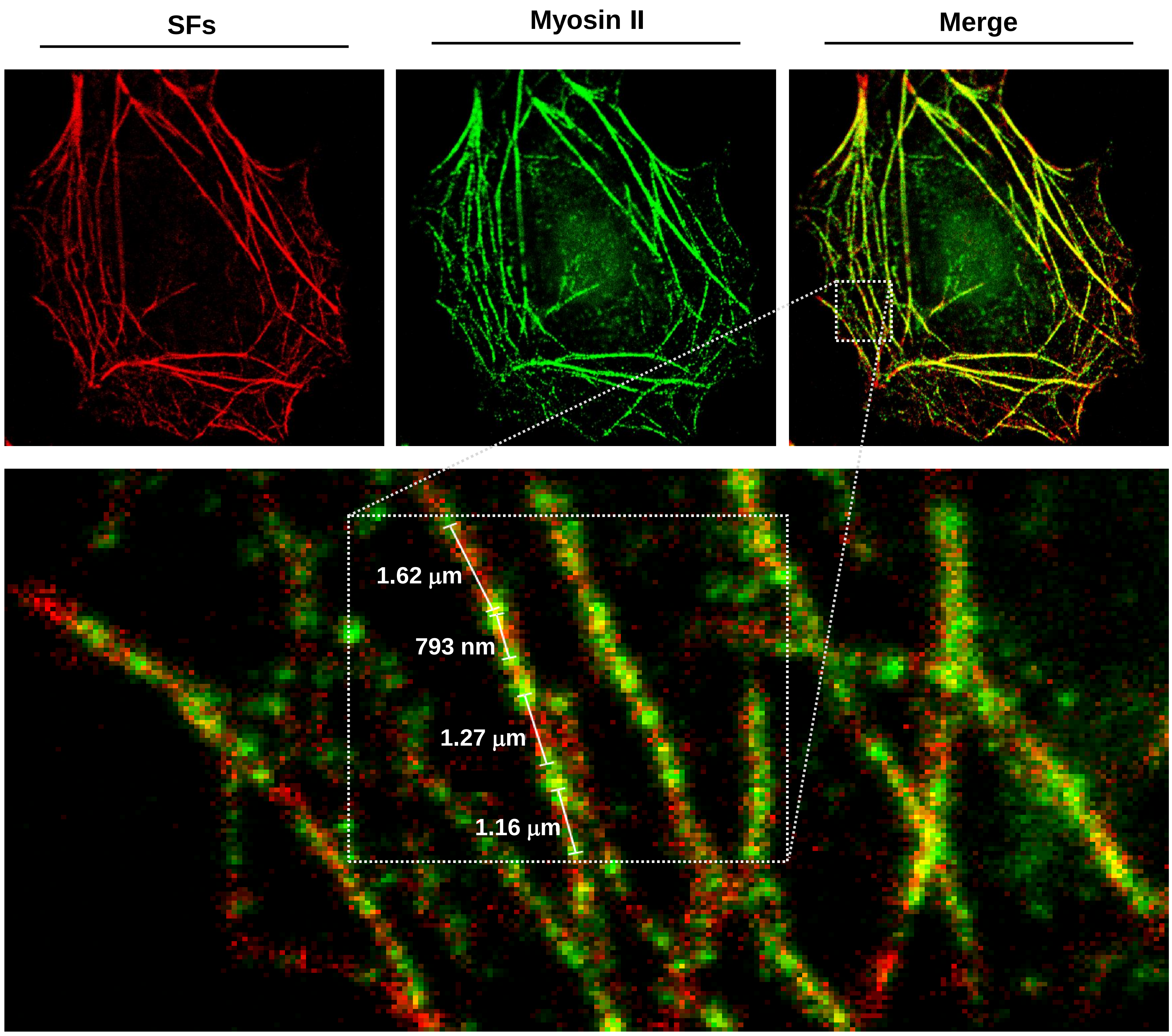

Figure S4

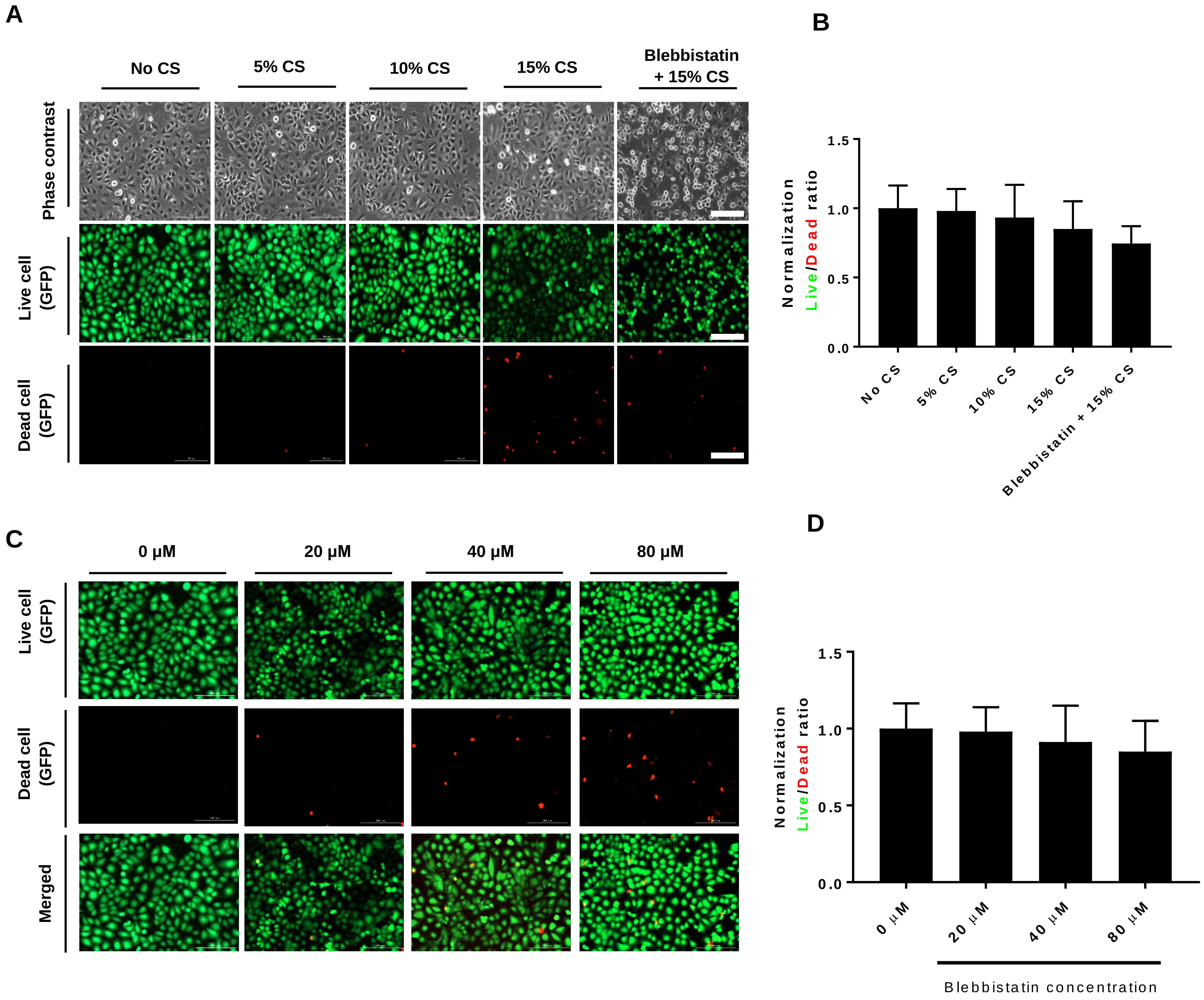
A
No CS
5% CS
10% CS
15% CS
Blebbistatin + 15% CS
Phase contrast
Live cell (GFP)
Dead cell (GFP)
B
Normalization Live/Dead ratio
1.5
1.0
0.5
0.0
No CS
5% CS
10% CS
15% CS
Blebbistatin + 15% CS
C
0 μM
20 μM
40 μM
80 μM
Live cell (GFP)
Dead cell (GFP)
Merged
D
Normalization Live/Dead ratio
1.5
1.0
0.5
0.0
0 μM
20 μM
40 μM
80 μM
Blebbistatin concentration

Figure S5

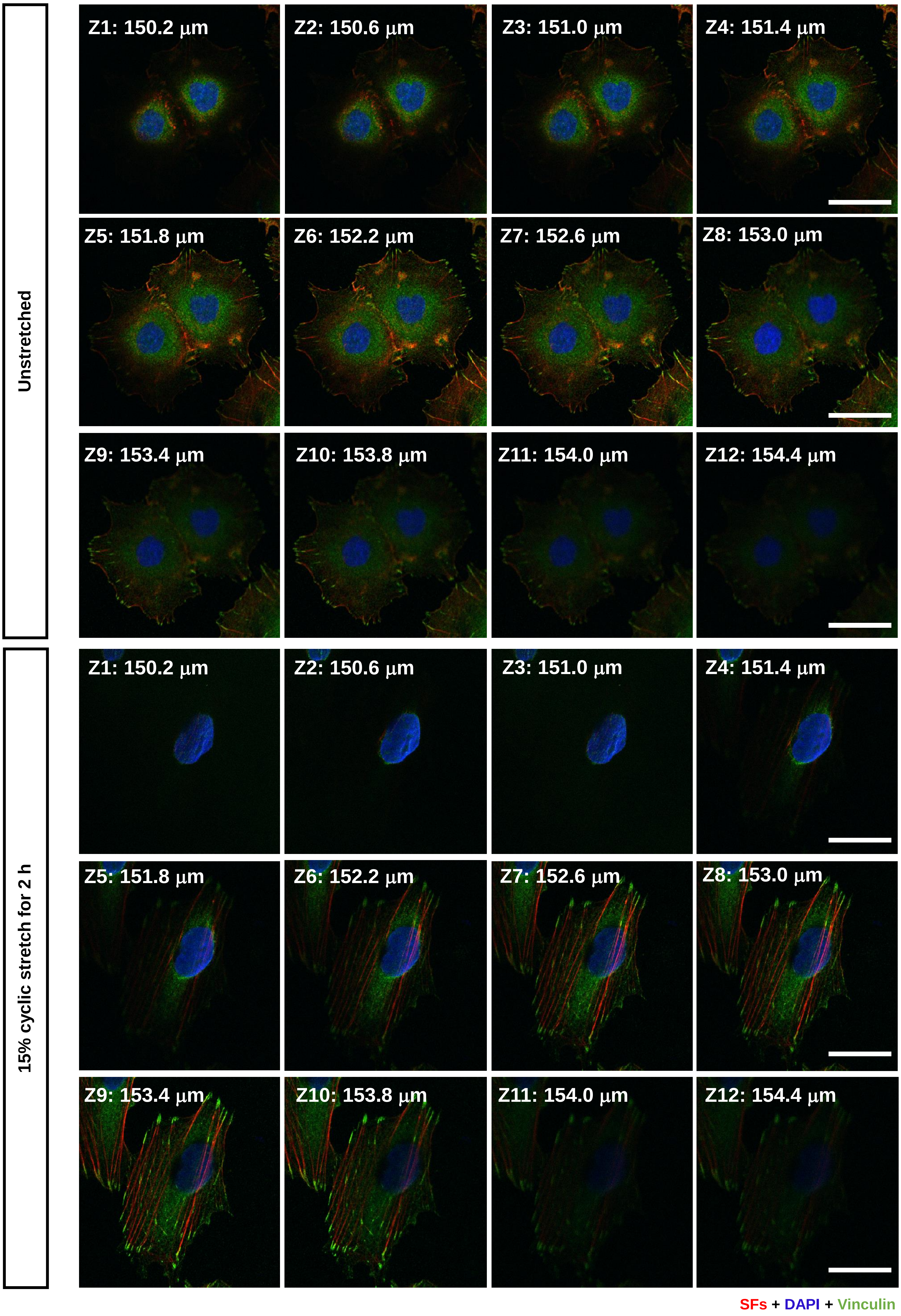
Unstretched
Z1: 150.2 μm
Z2: 150.6 μm
Z3: 151.0 μm
Z4: 151.4 μm
Z5: 151.8 μm
Z6: 152.2 μm
Z7: 152.6 μm
Z8: 153.0 μm
Z9: 153.4 μm
Z10: 153.8 μm
Z11: 154.0 μm
Z12: 154.4 μm
15% cyclic stretch for 2 h
Z1: 150.2 μm
Z2: 150.6 μm
Z3: 151.0 μm
Z4: 151.4 μm
Z5: 151.8 μm
Z6: 152.2 μm
Z7: 152.6 μm
Z8: 153.0 μm
Z9: 153.4 μm
Z10: 153.8 μm
Z11: 154.0 μm
Z12: 154.4 μm
SFs + DAPI + Vinculin

Figure S6

A

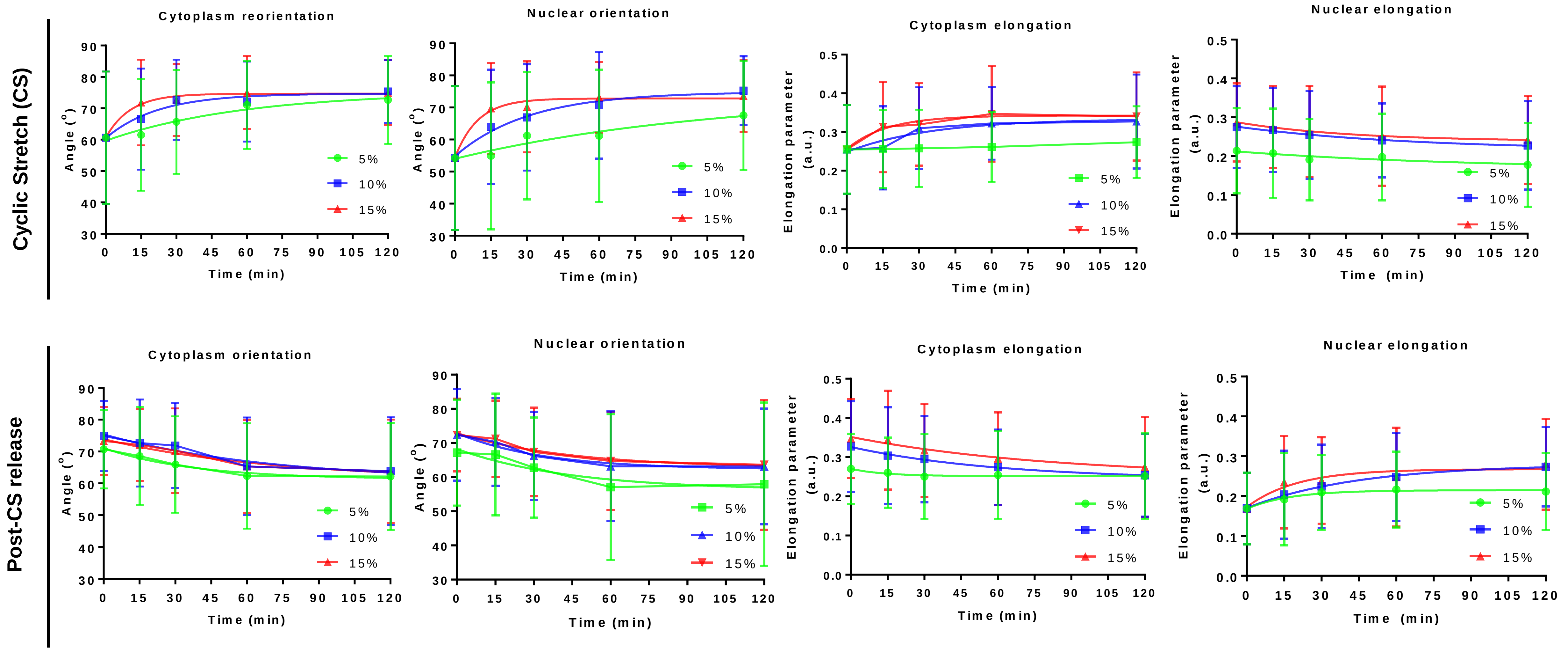

B

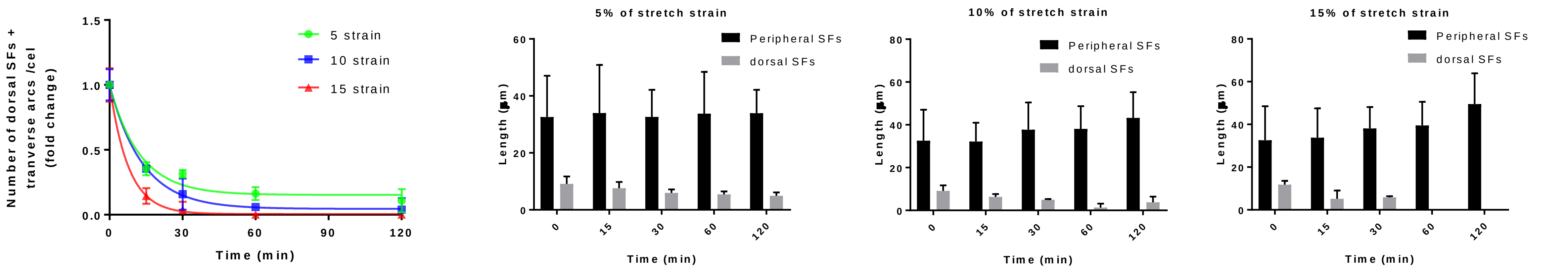

C

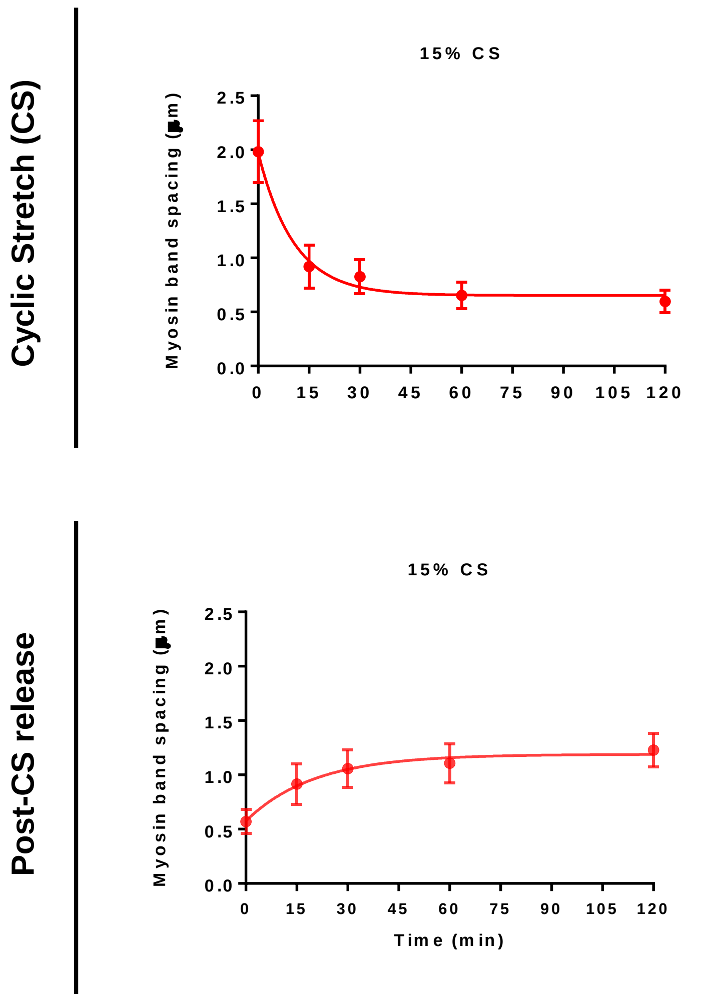

Figure S7

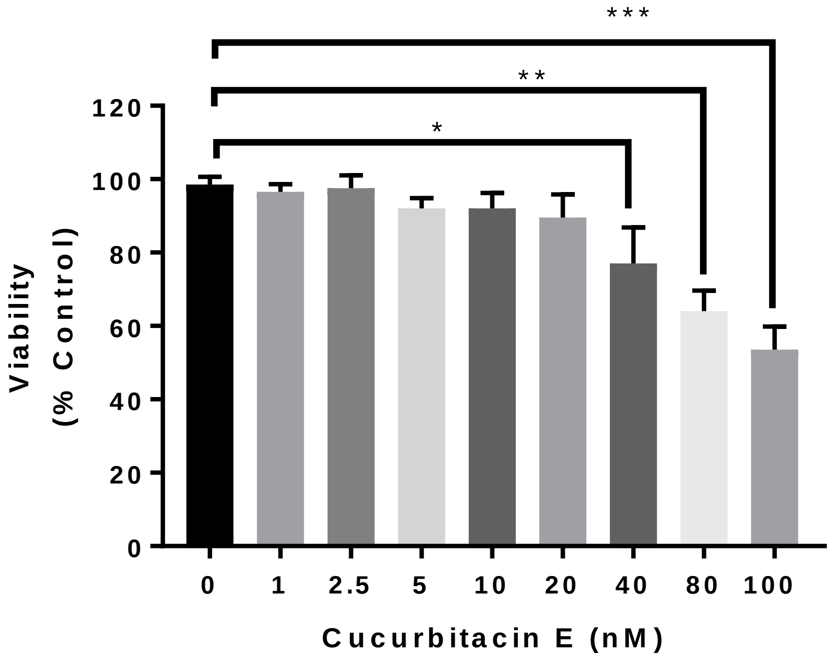
A
***
**
*
Viability
(% Control)
120
100
80
60
40
20
0
0 1 2.5 5 10 20 40 80 100
Cucurbitacin E (nM)

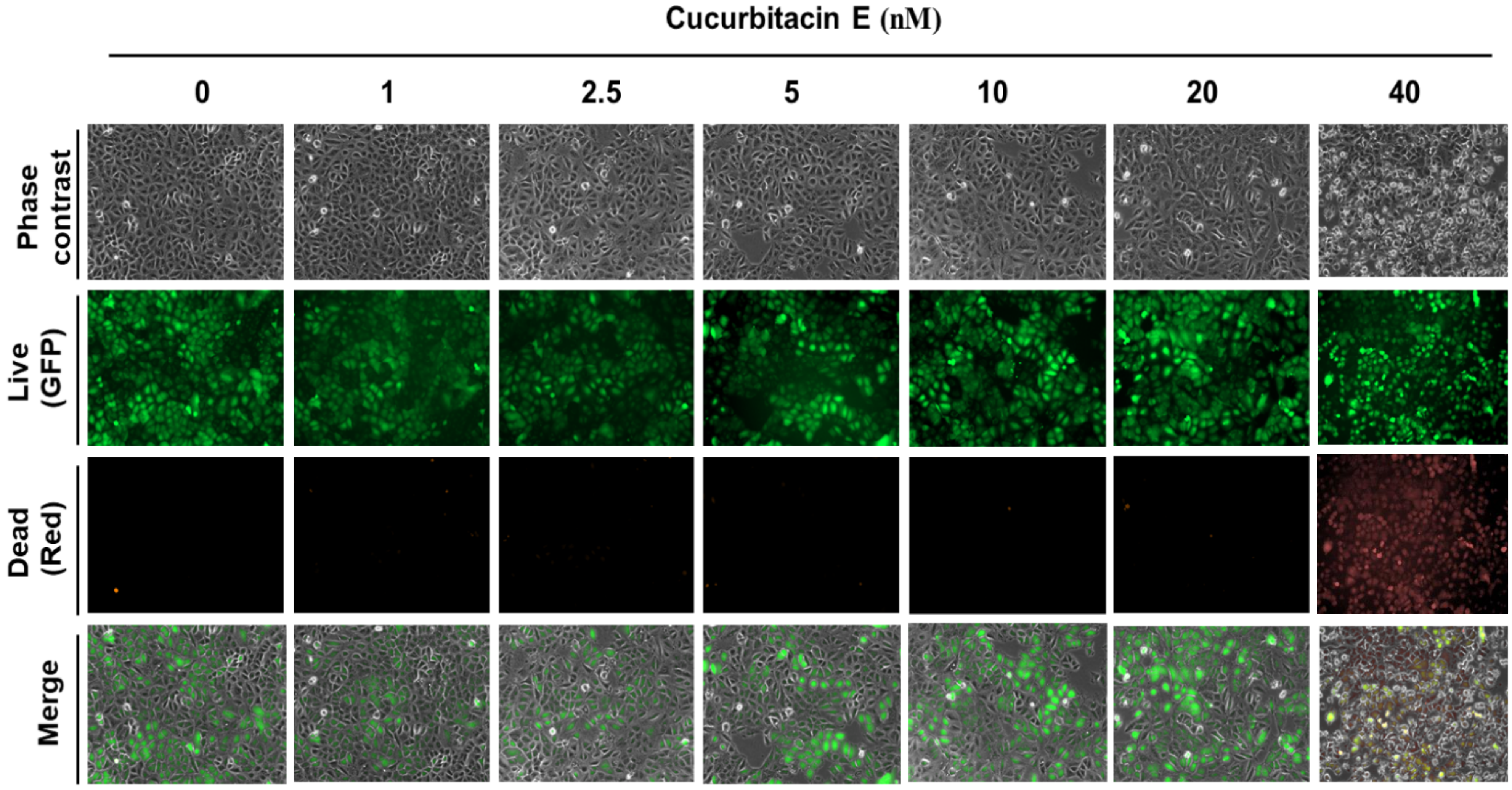
B
Cucurbitacin E (nM)
0 1 2.5 5 10 20 40
Phase contrast
Live (GFP)
Dead (Red)
Merge

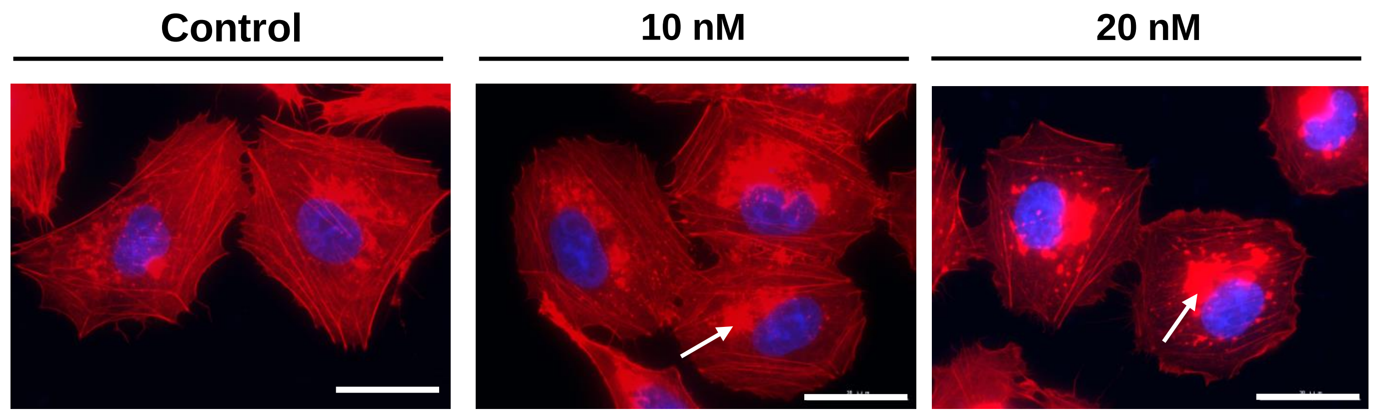
C
Control
10 nM
20 nM

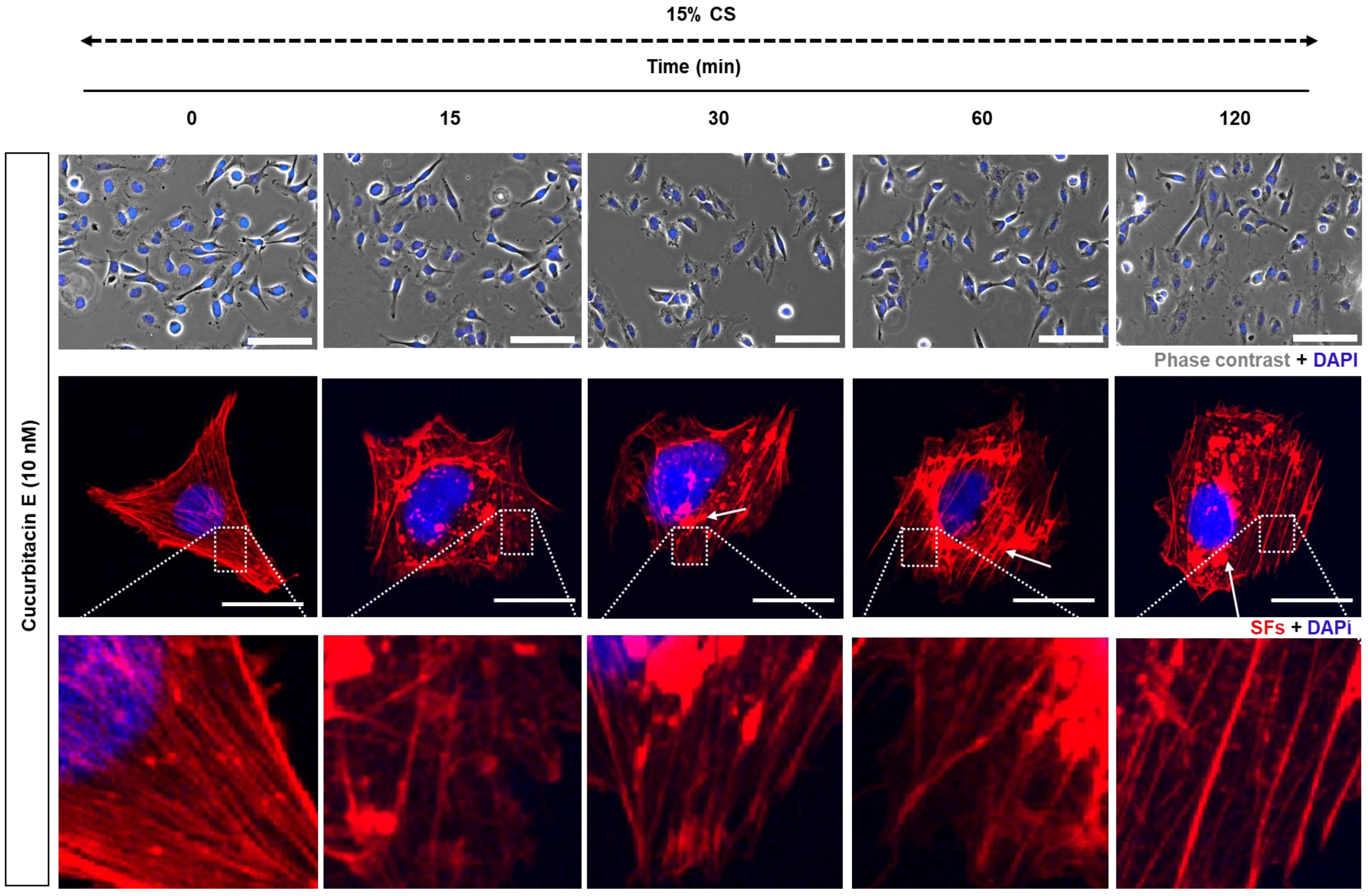
D
15% CS
Time (min)
0 15 30 60 120
Phase contrast + DAPi
SFs + DAPi
Cucurbitacin E (10 nM)

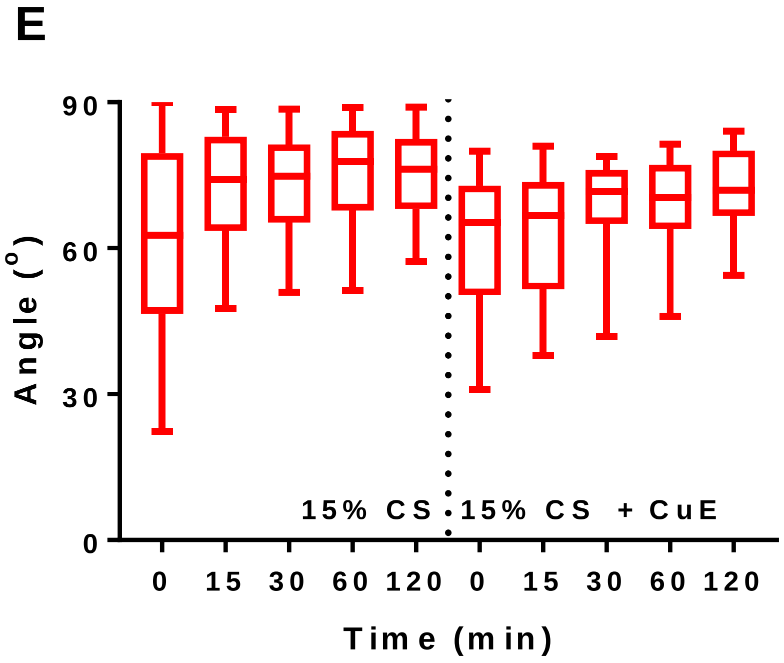
E
90
60
30
0
Angle (°)
15% CS
15% CS + CuE
0 15 30 60 120 0 15 30 60 120
Time (min)

Figure S8

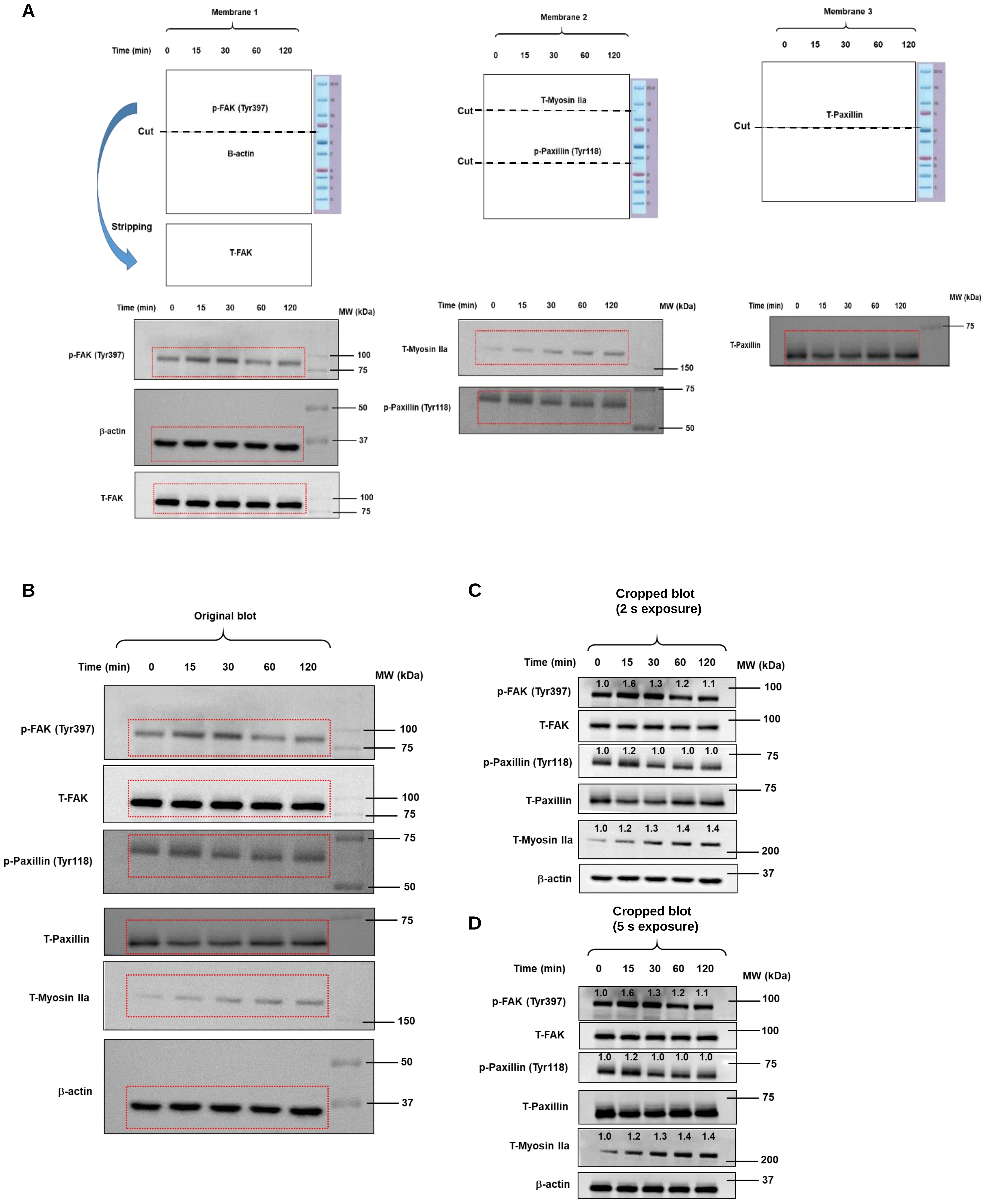
A
Membrane 1
Time (min) 0 15 30 60 120
p-FAK (Tyr397)
Cut
B-actin
Stripping
T-FAK
Membrane 2
0 15 30 60 120
T-Myosin IIa
Cut
p-Paxillin (Tyr118)
Cut
Membrane 3
0 15 30 60 120
T-Paxillin
Cut
Time (min) 0 15 30 60 120
MW (kDa)
p-FAK (Tyr397)
100
75
β-actin
50
37
T-FAK
100
75
Time (min) 0 15 30 60 120
MW (kDa)
T-Myosin IIa
150
p-Paxillin (Tyr118)
75
50
Time (min) 0 15 30 60 120
MW (kDa)
T-Paxillin
75
B
Original blot
Time (min) 0 15 30 60 120
MW (kDa)
p-FAK (Tyr397)
100
75
T-FAK
100
75
p-Paxillin (Tyr118)
75
50
T-Paxillin
75
T-Myosin IIa
150
β-actin
50
37
C
Cropped blot
(2 s exposure)
Time (min) 0 15 30 60 120
MW (kDa)
p-FAK (Tyr397)
1.0 1.6 1.3 1.2 1.1
100
T-FAK
100
p-Paxillin (Tyr118)
1.0 1.2 1.0 1.0 1.0
75
T-Paxillin
75
T-Myosin IIa
1.0 1.2 1.3 1.4 1.4
200
β-actin
37
D
Cropped blot
(5 s exposure)
Time (min) 0 15 30 60 120
MW (kDa)
p-FAK (Tyr397)
1.0 1.6 1.3 1.2 1.1
100
T-FAK
100
p-Paxillin (Tyr118)
1.0 1.2 1.0 1.0 1.0
75
T-Paxillin
75
T-Myosin IIa
1.0 1.2 1.3 1.4 1.4
200
β-actin
37

Figure S8

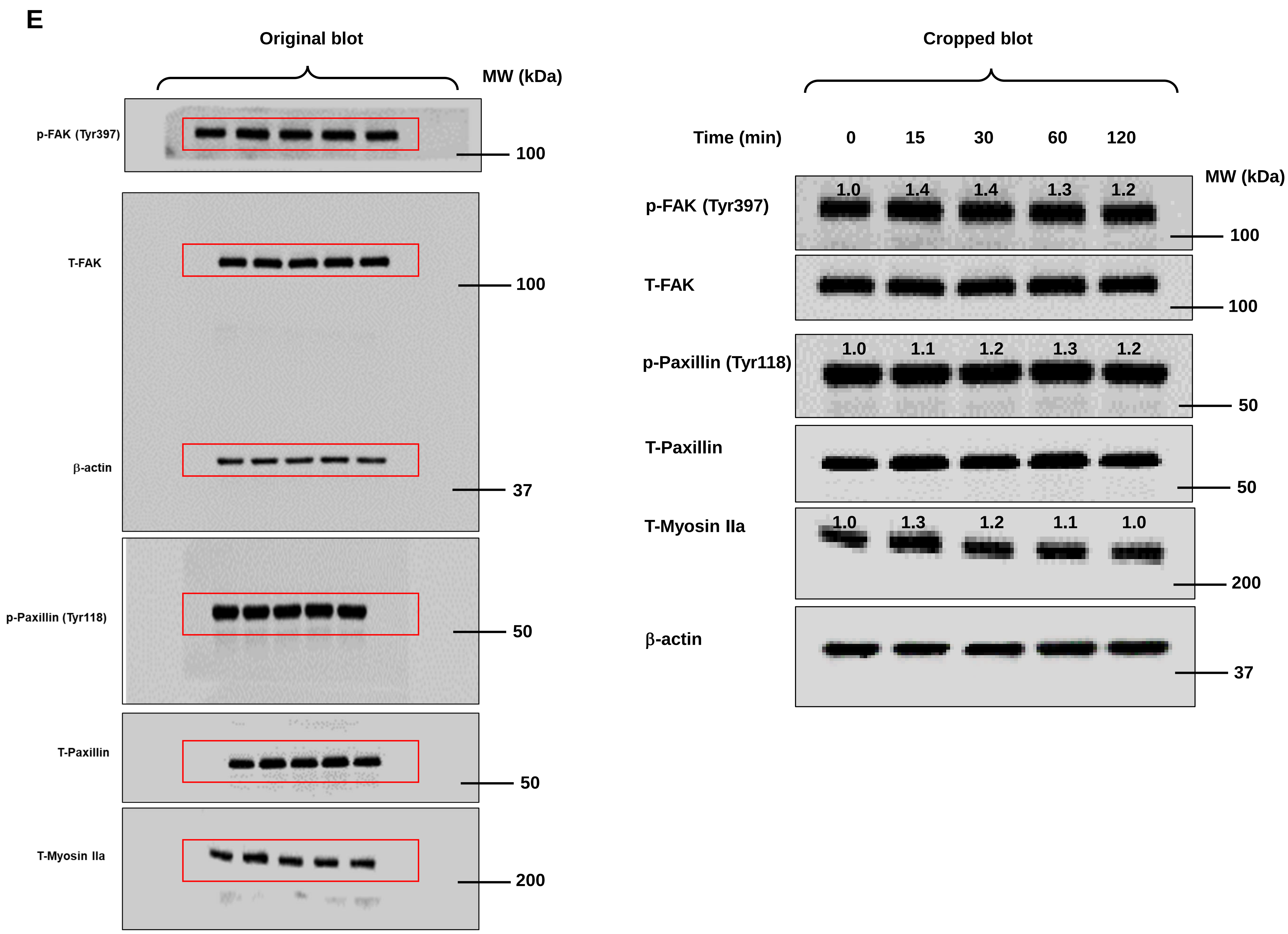
E
Original blot
MW (kDa)
p-FAK (Tyr397)
100
T-FAK
100
β-actin
37
p-Paxillin (Tyr118)
50
T-Paxillin
50
T-Myosin IIa
200
Cropped blot
Time (min)
0
15
30
60
120
MW (kDa)
p-FAK (Tyr397)
1.0
1.4
1.4
1.3
1.2
100
T-FAK
100
p-Paxillin (Tyr118)
1.0
1.1
1.2
1.3
1.2
50
T-Paxillin
50
T-Myosin IIa
1.0
1.3
1.2
1.1
1.0
200
β-actin
37
